# 5-LO inhibition ameliorates palmitic acid-induced ER stress, oxidative stress and insulin resistance via AMPK activation in murine myotubes

**DOI:** 10.1038/s41598-017-05346-5

**Published:** 2017-07-10

**Authors:** Hyun Jeong Kwak, Hye-Eun Choi, Hyae Gyeong Cheon

**Affiliations:** 1 Department of Pharmacology, Gachon University College of Medicine, Incheon, 21999 Republic of Korea; 2 Gachon Medical Research Institute, Gil Medical Center, Incheon, 21565 Republic of Korea

## Abstract

Leukotriene B4 (LTB4) production via the 5-lipoxygenase (5-LO) pathway contributes to the development of insulin resistance in adipose and hepatic tissues, but the role of LTB4 in skeletal muscle is relatively unknown. Here, the authors investigated the role of LTB4 in C2C12 myotubes in palmitic acid (PA)-induced ER stress, inflammation and insulin resistance. PA (750 μM) evoked lipotoxicity (ER stress, oxidative stress, inflammation and insulin resistance) in association with LTB4 production. 5-LO inhibition reduced all the lipotoxic effects induced by PA. On the other hand, PA did not induce cysteinyl leukotrienes (CysLTs), which themselves had no effect on ER stress and inflammation. The beneficial effects of 5-LO suppression from PA-induced lipotoxicity were related with AMPK activation. In *ob/ob* mice, once daily oral administration of zileuton (50, 100 mg/kg) for 5 weeks improved insulin resistance, increased AMPK phosphorylation, and reduced LTB4 and ER stress marker expression in skeletal muscle. These results show that 5-LO inhibition by either zileuton or 5-LO siRNA protects C2C12 myotubes from PA-induced lipotoxicity, at least partly via AMPK activation, and suggest that the *in vivo* insulin-sensitizing effects of zileuton are in part attributable to its direct action on skeletal muscle via LTB4 downregulation followed by AMPK activation.

## Introduction

The incidences of metabolic diseases, including type 2 diabetes mellitus (T2DM), continue to dramatically increase worldwide, possibly due to the obesity epidemic. Insulin resistance appears to be a key factor of obesity-driven T2DM, and leads to impairments of the action of insulin in target tissues, such as, fat, liver and skeletal muscle^1–3^. Skeletal muscle is the most important tissue in terms of insulin-mediated glucose disposal^4^, and thus, defects in insulin-induced glucose uptake by skeletal muscle are strongly linked to insulin resistance. A number of authors have suggested a relationship between skeletal muscle insulin resistance and the pathogenesis of T2DM, based on the observation that T2DM patients exhibit reduced insulin sensitivity of skeletal muscle^4, 5^.

Endoplasmic reticulum (ER) is the major site for the synthesis, folding, and trafficking of secretory and membrane proteins^6^, and is highly sensitive to redox status^7, 8^. Recently, ER stress has been suggested to importantly contribute to the development of insulin resistance, which is sensed by a number of factors, such as, inositol-requiring enzyme-1α (IRE-1α), protein kinase RNA-like endoplasmic reticulum kinase (PERK), and activating transcription factor-6 (ATF-6), and leads to translational attenuation and cellular dysfunction^9^. Several reports have shown that chemical inhibitions of oxidative stress or ER stress improve insulin sensitivity and glucose homeostasis in the skeletal muscle of obese patients^7, 10, 11^, which suggests oxidative and ER stress be considered major targets for combating insulin resistance.

Various inflammatory mediators, such as, cytokines, eicosanoids, and other factors, are also linked with insulin resistance^12, 13^. The 5-LO generates leukotrienes (LTs) via two step lipoxygenation of arachidonic acid. Once formed by 5-LO, unstable epoxide LTA4 is transformed either to LTB4 or to a cysteinyl leukotriene LTC4, subsequently LCD4 and LTE4 through glutathione conjugation^14^. Among 5-LO products, LTB4 is a potent chemotactic factor, and is produced by adipocytes and stimulates macrophage infiltration into adipose tissues via BLT1 receptor^15, 16^. Others have reported hepatic lipid accumulation and inflammation were induced by LTB4 produced directly by hepatocytes or indirectly delivered from adipose tissue^17, 18^. Further support for the role of LTB4 in obesity-induced insulin resistance was provided by animal studies on genetically modified mice. For example, deficiency of either 5-LO or BLT1 receptor improved insulin resistance in a diet-induced obese mouse model^19^. Similarly, pharmacological modulation of LTB4 using zileuton or a BLT1 receptor antagonist protected high fat diet-induced mice from insulin resistance^15^. However, while the influences of LTB4 on adipose tissue, liver inflammation, and insulin resistance have been extensively studied, comparatively little is known of the effects of LTB4 production on skeletal muscle. With regard to 5-LO-LTB4 pathway in skeletal muscle, 5-LO expression and LTB4 production was detected in human and rodent muscle, indicating the existence of functional pathway in muscle tissue^20, 21^.

To this end, we investigated the effects of 5-LO inhibition by either zileuton or 5-LO siRNA on PA-induced ER stress and insulin resistance in C2C12 myotubes to assess the role of LTB4 in skeletal muscle.

## Results

### 5-LO pathway is involved in PA-induced ER stress and oxidative stress

**Figure 1:**
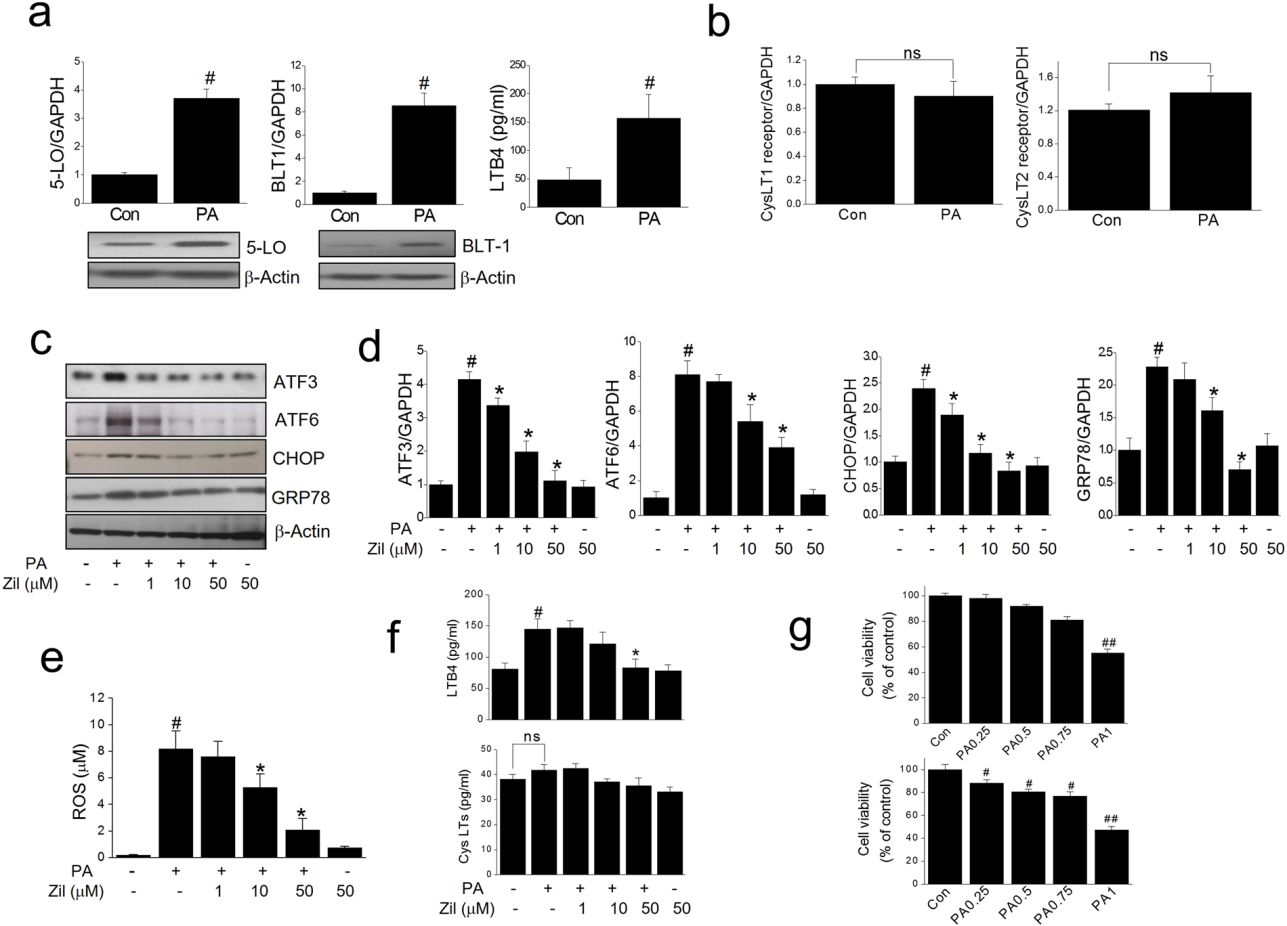
Effects of zileuton on PA-induced ER stress and oxidative stress. The mRNA expressions of 5-LO, BLT1, CysLT1 and CysLT2 in C2C12 myotubes were determined by qPCR with or without PA treatment (750 μM for 12 h) (**a**, upper). 5-LO and BLT1 protein levels were determined by western blotting with or without PA treatment (**a**, lower). LTB4 production was determined by LTB4 kits, with or without PA treatment (750 μM for 24 h) (**a**). The mRNA expressions of CysLT1 and 2 receptors were determined by qPCR with or without PA treatment (**b**). C2C12 myotubes were pre-exposed to different concentrations of zileuton (1-50 μM) for 1 h and then treated with PA (750 μM) in the presence of zileuton for 12 h (qPCR) or 24 h (western blot, LTB4/CysLTs ELISA and ROS). The expression levels of ER stress markers were then determined by western blotting (**c**) and qPCR (**d**). ROS production was assessed using AmplexRed (**e**), and LTB4 and CysLTs production was determined by LTB4 and CysLTs ELISA kits, respectively (**f**). Cell viability was determined after 24 h PA treatment by MTT assay (**g**, upper) and LDH release measurement (**g**, lower). The results of the western blotting are shown as the representative of three independent experiments (**a** and **c**), and other results are expressed as the means ± SDs of three experiments performed in triplicate. ^#^ *P* < 0.05, ^##^ *P* < 0.01 *vs*. non-treated controls, *P < 0.05 *vs*. PA alone. ns; not significant.

Initially, we assessed the expressions of 5-LO and BLT1 receptor in C2C12 myotubes. As shown in Fig. 1a, mRNA expression of 5-LO and BLT1 was confirmed, and correspondingly the production of LTB4 was detected and increased by PA (750 μM) as were the expressions of 5-LO and BLT1 protein, indicating the 5-LO-LTB4 pathway is constitutively expressed and functional in C2C12 cells. On the other hand, mRNA expressions of CysLT1 and 2 receptors were unchanged by PA (Fig. 1b).

Next, to evaluate the role of LTB4 in PA-induced ER stress, the effects of zileuton on PA-induced ER stress were examined in myotubes. Zileuton reduced the PA-induced expression of ER stress markers (Fig. 1c and d) and ROS levels (Fig. 1e) in a concentration dependent manner, and the almost complete inhibition to control level was achieved at 50 μM. To validate the effects of zileuton were related with 5-LO inhibition, we measured the LTB4 production under these conditions, which revealed that zileuton indeed reduced LTB4 production, but little effect on CysLT levels (Fig. 1f). Zileuton was found to have no cytotoxic effect at concentrations up to 50 μM (results not shown), and PA at 750 μM reduced cell viability approximately to 80% of control (Fig. 1g upper: MTT assay, 1 g lower: LDH assay). These results suggest that LTB4 rather than CysLTs is involved in PA-induced ER stress in C2C12 cells.

### 5-LO pathway is involved in PA-induced inflammation and insulin resistance

**Figure 2:**
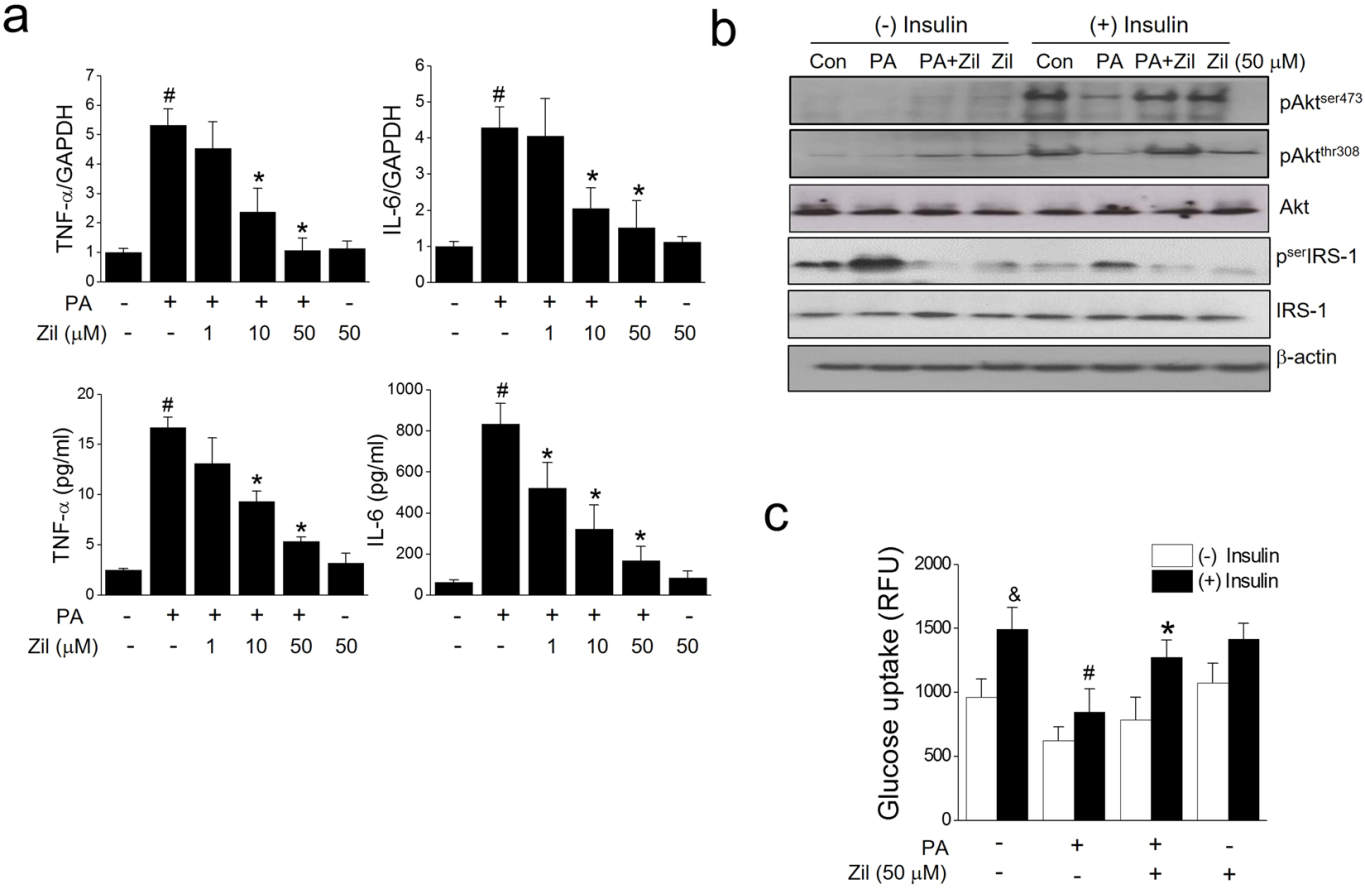
Effects of zileuton on PA-induced inflammation and insulin resistance. Differentiated C2C12 myotubes were exposed to different concentrations of zileuton (1–50 μM) for 1 h and then treated with PA (750 μM) in the presence of zileuton for 12 h (qPCR) or 24 h (ELISA). Levels of pro-inflammatory cytokines were determined by ELISA and qPCR (**a**). After 24 h of PA treatment, insulin (1 μg/ml) was added for 30 min, and then insulin signaling, as indicated by Akt and serIRS-1 phosphorylation, was assessed by western blotting (**b**). Glucose uptake was determined by 2-NBDG (**c**). The results of the western blotting are shown as the representative of three independent experiments (**b**), and other results are expressed as the means ± SDs of three experiments performed in triplicate. ^#^ *P* < 0.05 *vs*. non-treated controls, *P < 0.05 *vs*. PA alone, ^&^ *P* < 0.05 *vs*. (−) insulin.

Since LTB4 is a well-known mediator of inflammatory response, we investigated whether zileuton could prevent PA-induced inflammation in C2C12 myotubes. As was expected, zileuton pre-treatment decreased the PA-induced productions of TNF-α and IL-6 (Fig. 2a), and ameliorated PA-induced impairments of the insulin signaling pathway, *i*.*e*., reduced serIRS-1 phosphorylation and increased Akt phosphorylation (Fig. 2b). Accordingly, insulin-stimulated glucose uptake was decreased by PA (59.4% reduction), but zileuton (50 μM) almost completely blocked this effect of PA (Fig. 2c). Zileuton itself had little effect on either basal or insulin-stimulated glucose uptake.

### LTB4 is a mediator of PA-induced lipotoxicity

**Figure 3:**
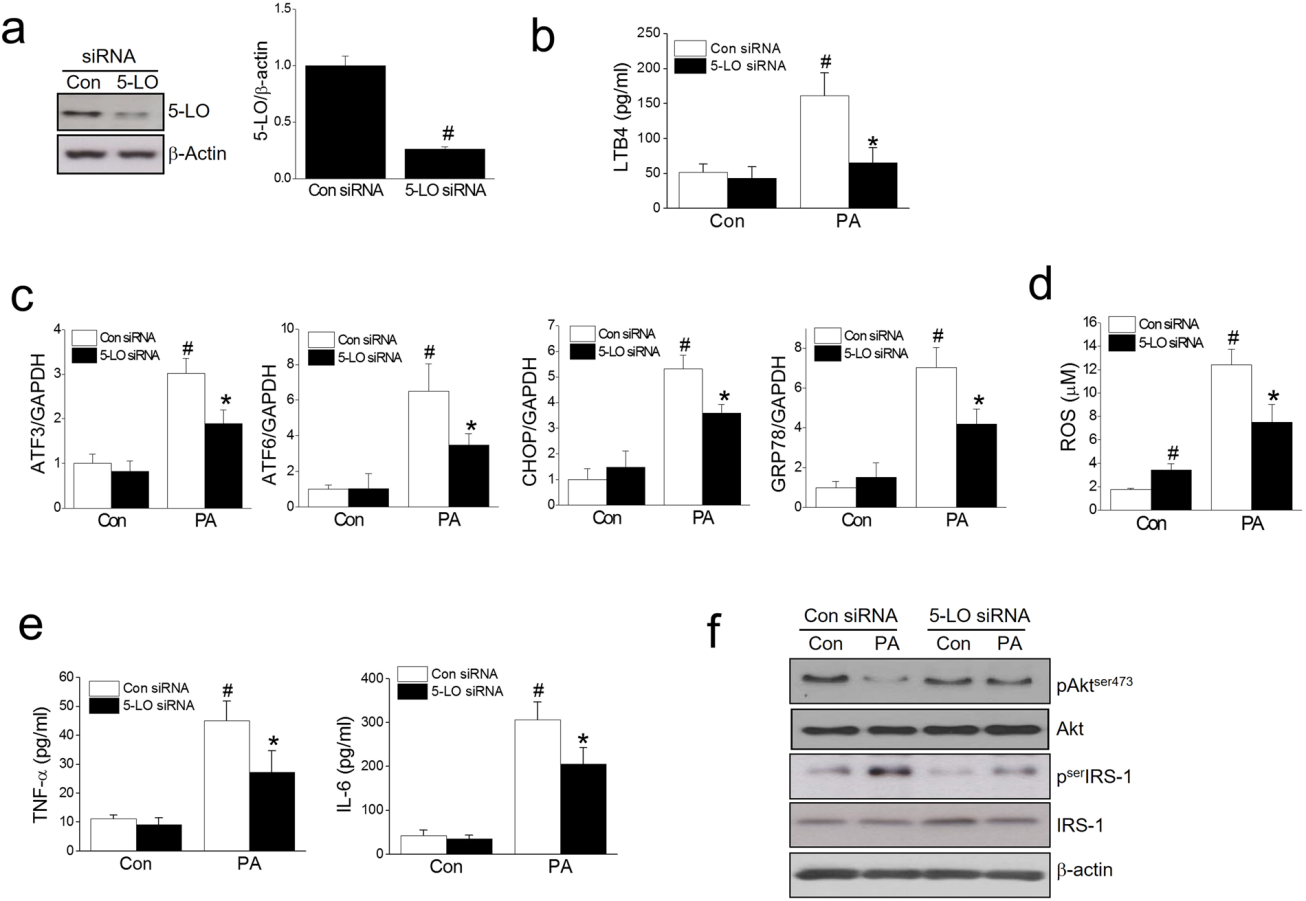
Effects of 5-LO siRNA on PA-induced lipotoxicity. C2C12 myotubes were transfected with negative control siRNA or 5-LO siRNA (20 nM), and then treated with PA (750 μM) for 12 h (qPCR) or 24 h (western blot, ELISA, ROS). The effects of 5-LO knockdown were determined by western blotting (**a**). Levels of LTB4 in culture media were measured by ELISA (**b**). The effects of 5-LO siRNA on the mRNA levels of ER stress markers were determined by qPCR (**c**). ROS production was assessed using AmplexRed (**d**). Levels of pro-inflammatory cytokines were determined by ELISA (**e**). Akt and serIRS-1 phosphorylation was assessed by western blotting (**f**). The results of the western blotting are shown as the representative of three independent experiments (**f**), and other results are expressed as means ± SDs of three experiments performed in triplicate. ^#^ *P* < 0.05 *vs*. non-treated control siRNA, *P < 0.05 *vs*. PA-treated control siRNA.

To confirm the role played by LTB4 in ER stress, oxidative stress, and insulin resistance, 5-LO was knocked down using 5-LO siRNA to 30% of the control level (Fig. 3a), and LTB4 production by PA was also markedly inhibited by 5-LO siRNA (Fig. 3b). Similar observation with zileuton, 5-LO siRNA reduced PA-induced ER stress marker expression, ROS production, proinflammatory cytokine expression, and restored PA-impaired insulin signaling (Fig. 3c–f).

**Figure 4:**
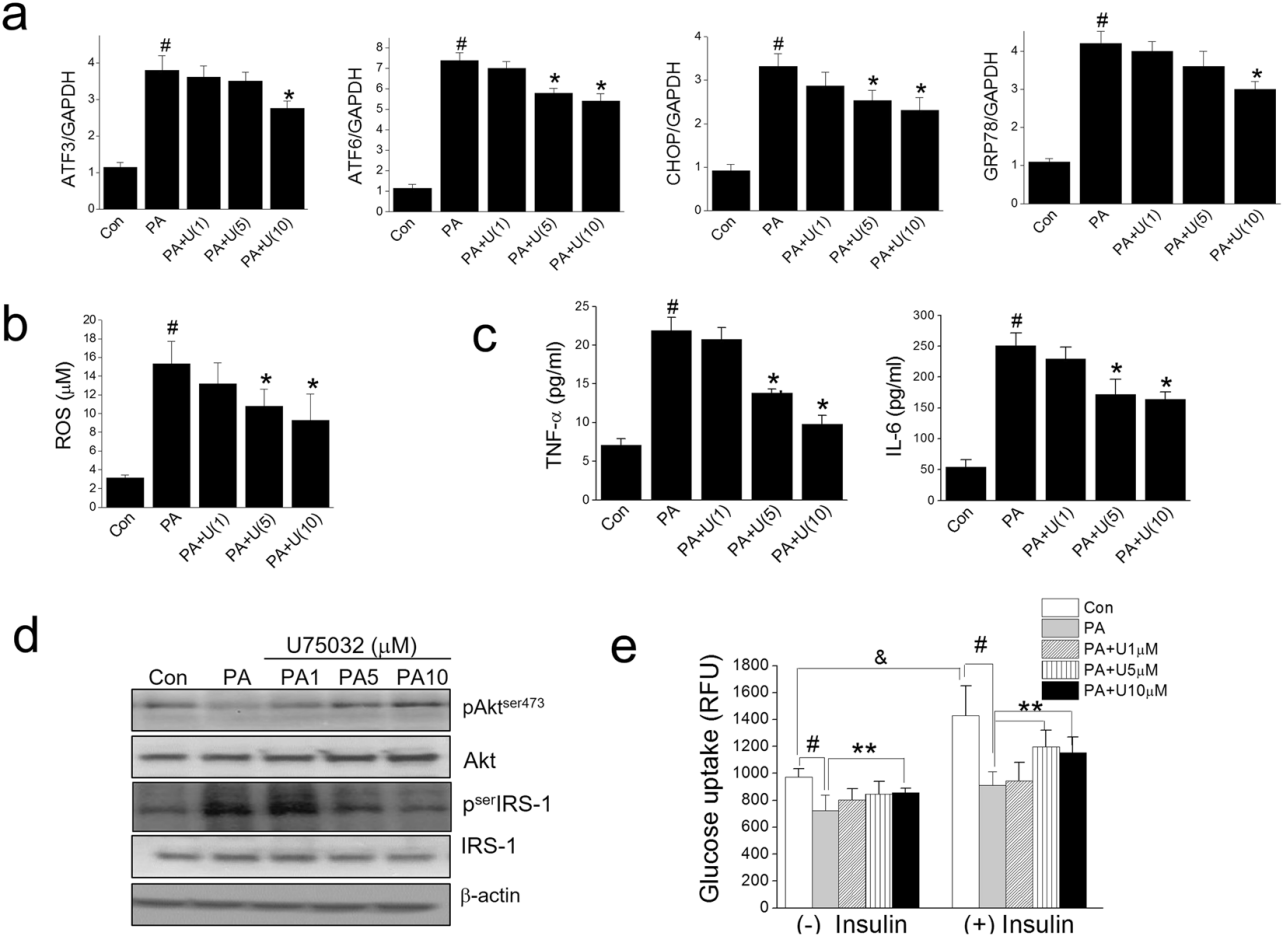
Effects of BLT1 receptor antagonist, U75032 on PA-induced lipotoxicity. Differentiated C2C12 myotubes were exposed to different concentrations of U75032 (1, 5, 10 μM) for 1 h and then treated with PA (750 μM) in the presence of U75032 for 12 h (qPCR) or 24 h (ELISA). Levels of ER stress markers were determined by qPCR (**a**). ROS production was assessed using AmplexRed (**b**). Levels of pro-inflammatory cytokines were determined by ELISA (**c**). Akt and serIRS-1 phosphorylation was assessed by western blotting (**d**). After 24 h of PA treatment, insulin (1 μg/ml) was added for 30 min, and then glucose uptake was determined by 2-NBDG (**e**). The results of the western blotting are shown as the representative of three independent experiments (**d**), and other results are expressed as the means ± SDs of three experiments performed in triplicate. ^#^ *P* < 0.05 *vs*. non-treated controls, *P < 0.05, **P < 0.01 *vs*. PA alone, ^&^ *P* < 0.05 *vs*. (−) insulin.

In line with the inhibition of LTB4 production by zileuton, we determined the effects of BLT1 receptor antagonist against PA-induced lipotoxicity in myotube. U75032, a BLT1 receptor antagonist concentration dependently reduced ER stress, ROS and inflammation in association with enhanced insulin signaling and glucose uptake (Fig. 4). These results further support the hypothesis that PA-induced LTB4 production is a critical player in the ER stress, ROS, inflammation, insulin resistance in myotube.

**Figure 5:**
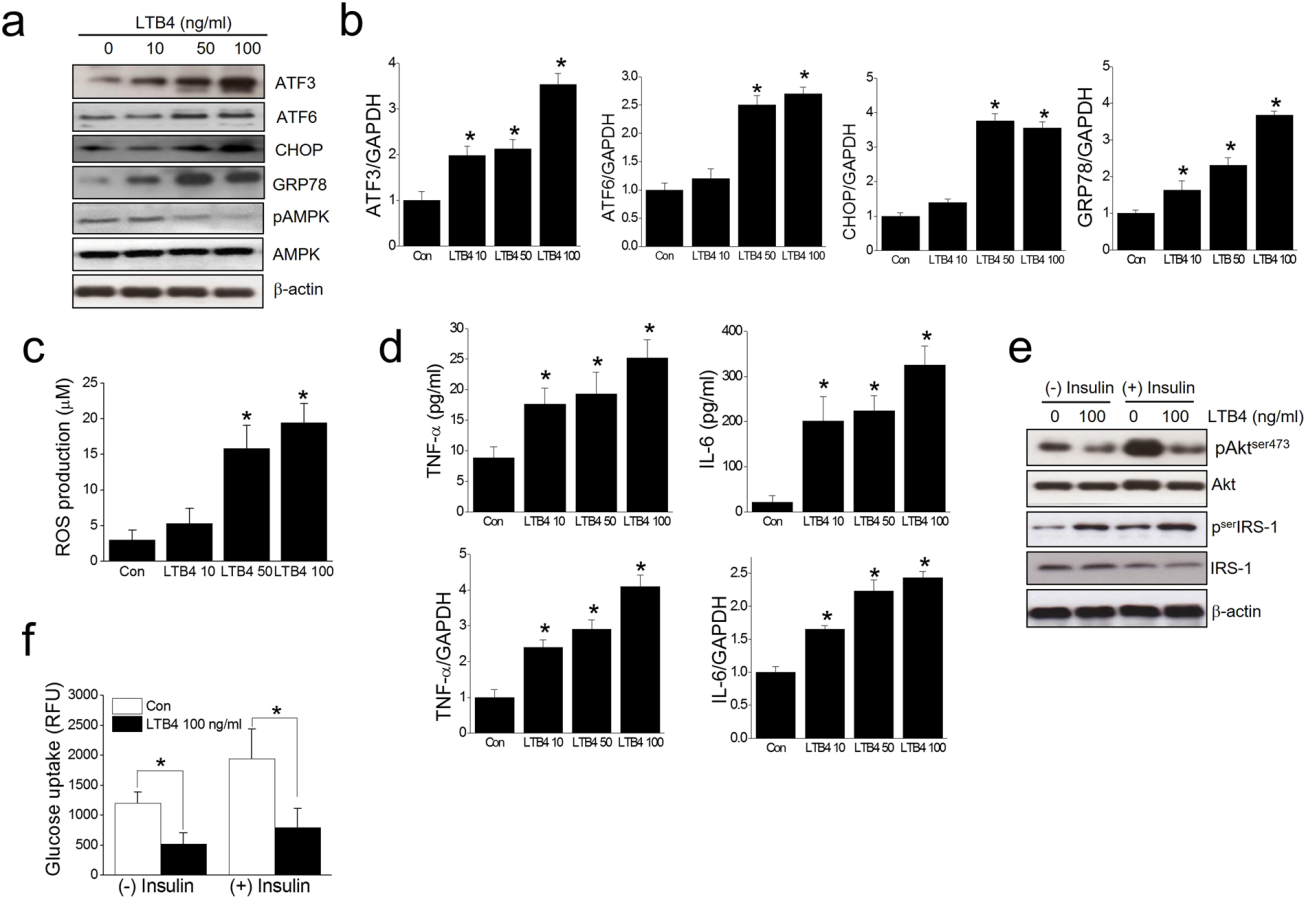
Effects of LTB4 on ER stress marker, oxidative stress, inflammation and insulin signaling. C2C12 myotubes were exposed to LTB4 (0–100 ng/ml) for 12 h (qPCR) or 24 h (western blot, ELISA, ROS, glucose uptake). The expression levels of ER stress markers were then determined by western blotting (**a**) and qPCR (**b**). ROS production was assessed using AmplexRed (**c**). Levels of pro-inflammatory cytokines were determined by ELISA and qPCR (**d**). Akt and serIRS-1 phosphorylation was assessed by western blotting (**e**), and glucose uptake was determined by 2-NBDG in the presence or absence of insulin (1 μg/ml) for 30 min (**f**). The results of the western blotting are shown as the representative of three independent experiments (**a**), and other results are expressed as the means ± SDs of three experiments performed in triplicate. *P < 0.05 *vs*. control.

To further determine the role of LTB4 in C2C12 myotubes, we examined the direct effects of LTB4 on C2C12 cells. LTB4 concentration dependently increased the expression of ER stress markers, ROS production, proinflammatory markers, impaired insulin signaling and glucose uptake, as shown in Fig. 5a–f. On the contrary, all cysLTs (*i*.*e*. LTC4, LTD4 and LTE4) had little effect on the expression of ER stress, proinflammatory markers, insulin signaling and glucose uptake (Supplementary Fig. 1). These results suggest that LTB4 produced by PA may act, at least partly, to induce ER stress, oxidative stress, inflammation, and insulin resistance possibly in an autocrine fashion.

### AMPK activation is involved in the protective effects of 5-LO inhibition

**Figure 6:**
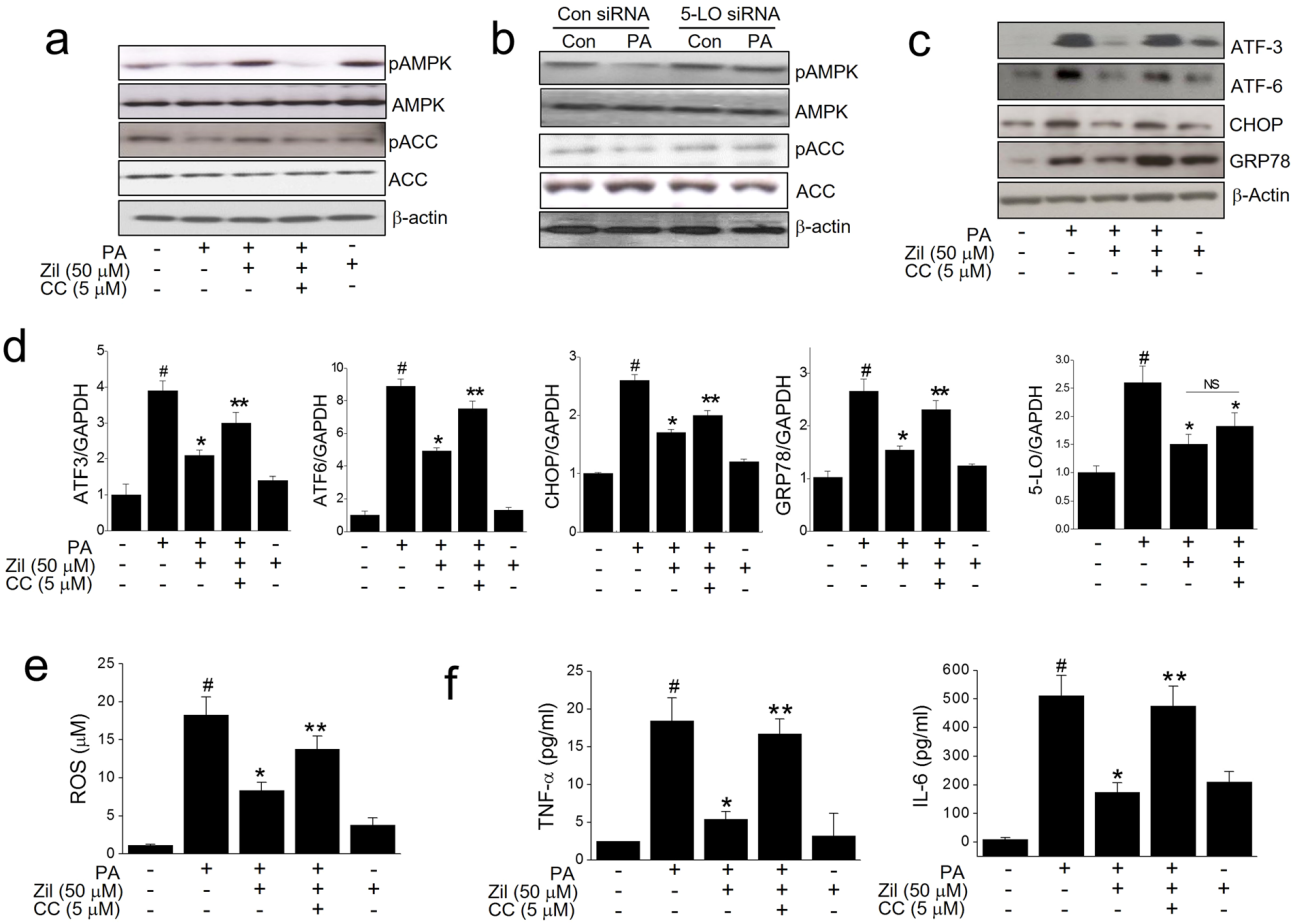
Effects of compound C on the zileuton action. C2C12 myotubes were exposed to 50 μM zileuton for 1 h in the absence or presence of 5 μM compound C, and then treated with PA (750 μM) for 12 h (qPCR) or 24 h (western blot, ROS and ELISA). Phosphorylated AMPK and ACC levels were determined by western blotting (**a**). 5-LO siRNA transfected C2C12 myotubes were treated with PA (750 μM) for 24 h, and the phosphorylated AMPK and ACC levels were determined by western blotting (**b**). The expression levels of ER stress markers (**c** and **d**) and 5-LO (**d**) were determined by western blotting (**c**) and qPCR (**d**) after cotreatment of zileuton and compound C followed by PA treatment. ROS production was assessed using AmplexRed (**e**). Levels of TNF-α and IL-6 were determined by ELISA (**f**). The results of the western blotting are shown as the representative of three independent experiments (**a–c**), and other results are expressed as the means ± SDs of three experiments performed in triplicate. ^#^ *P* < 0.05 *vs*. non-treated controls, *P < 0.05 *vs*. PA alone, and **P < 0.05 *vs*. PA plus zileuton.

AMPK activation is a known key mediator of myotube insulin sensitization. Since LTB4 appears to suppress AMPK activation^22^, we examined its effect on phosphorylated AMPK levels, and confirmed that LTB4 concentration dependently reduced AMPK phosphorylation as shown in Fig. 5a. Likewise, PA reduced pAMPK and pACC levels, which was reversed by pre-treatment with either zileuton or 5-LO siRNA (Fig. 6a and b). On the other hand, cotreatment of zileuton with compound C (5 µM) in the presence of PA significantly inhibited the effects of zileuton on AMPK and ACC phosphorylation, ER stress markers, ROS production, and inflammatory cytokines induced by PA (Fig. 6a,c–f), suggesting that AMPK activation critically underlies the protective action of zileuton against PA-induced lipotoxicity in C2C12 myotubes. Interestingly, the effects of zileuton and 5-LO siRNA on basal AMPK phosphorylation appear to be different, in that zileuton, but not 5-LO siRNA markedly enhanced basal AMPK phosphorylation (Fig. 6a and b), implying that other mechanisms besides 5-LO inhibition may exist for zileuton-induced AMPK phosphorylation in basal condition.

**Figure 7:**
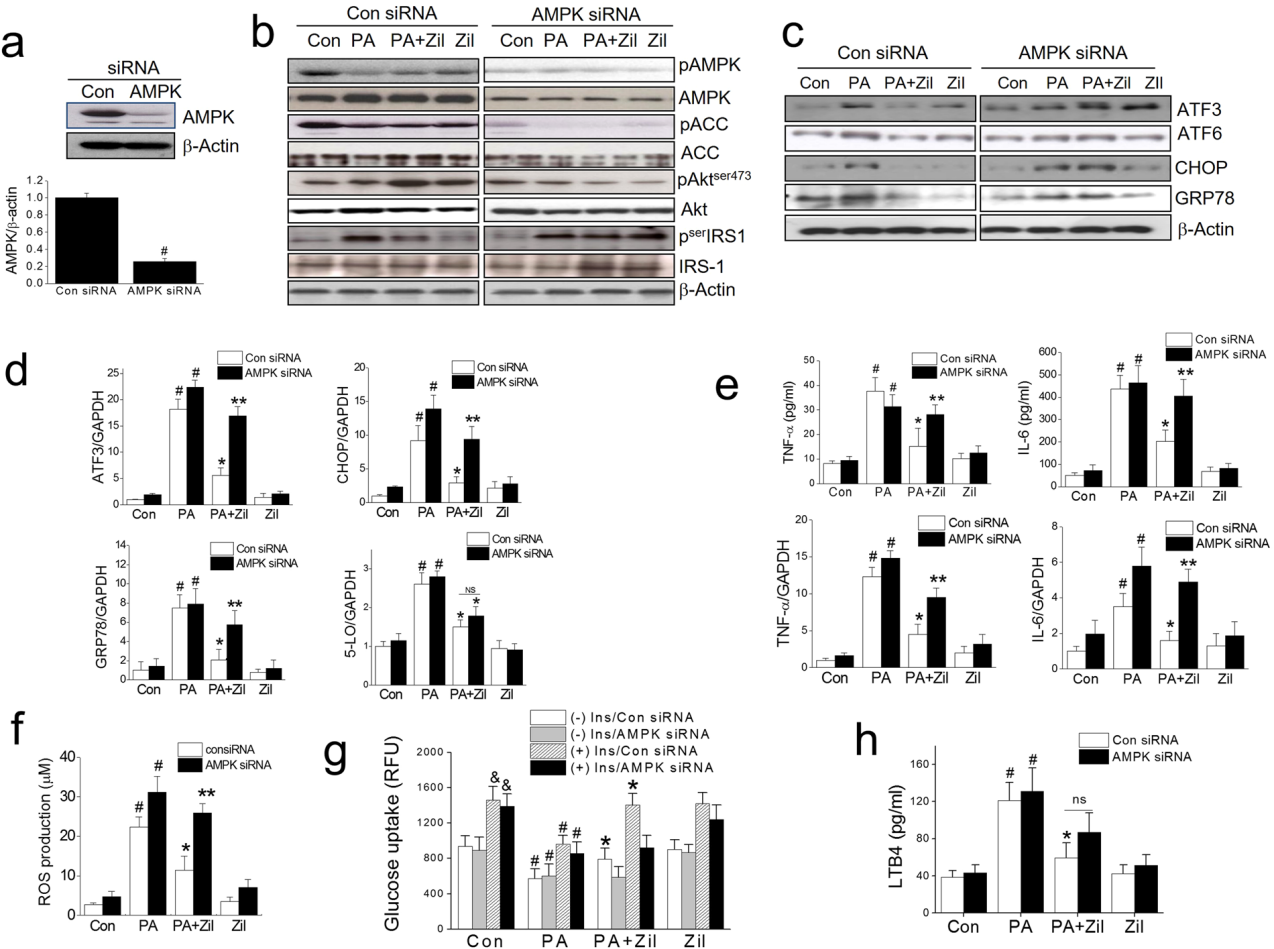
The effect of AMPK knockdown on the zileuton action. C2C12 myotubes were transfected with negative control siRNA or AMPK siRNA (20 nM), treated with 50 μM zileuton for 1 h, and then with PA (750 μM) for 12 h (qPCR) or 24 h (western blot, ELISA, ROS, and glucose uptake). The effect of AMPK knockdown was determined by western blotting (**a**). The effects of AMPK siRNA on Akt/serIRS-1 phosphorylation, and AMPK/ACC phosphorylation (**b**) and ER stress markers were determined by western blotting (**c**) and qPCR (**d**). The expression of 5-LO was determined by qPCR (**d**). Cellular TNF-α and IL-6 mRNA levels and secreted TNF-α and IL-6 levels were determined by qPCR and ELISA (**e**). ROS production was assessed using AmplexRed (**f**). Glucose uptake was determined by 2-NBDG in the presence or absence of insulin (1 μg/ml) for 30 min (**g**). LTB4 production was measured by ELISA kits (**h**). The results of the western blotting are shown as the representative of three independent experiments (**b** and **c**), and other results are expressed as the means ± SDs of three experiments performed in triplicate. ^#^ *P* < 0.05 *vs*. non-treated controls, *P < 0.05 *vs*. PA alone, **P < 0.05 *vs*. control siRNA of PA plus zileuton, ^&^ *P* < 0.05 *vs*. (−) insulin, ns; not significant.

To confirm the involvement of AMPK in the effects of zileuton, we examined the effects of AMPK siRNA (20 nM; targeting the α catalytic subunit). AMPK siRNA reduced AMPK expression by up to 25% of control in C2C12 myotubes (Fig. 7a). The effects of zileuton on Akt phosphorylation and serIRS-1 phosphorylation disappeared in AMPK knockdowned cells (Fig. 7b). Likewise, AMPK knockdown abolished the protective effects of zileuton on ER stress marker expression, proinflammatory cytokine expression, ROS production, and insulin-stimulated glucose uptake (Fig. 7c–g). On the contrary, the suppression of LTB4 production by zileuton was not reversed by AMPK siRNA (Fig. 7h). Furthermore, reduced 5-LO mRNA expression by zileuton was unaffected by either compound C or AMPK siRNA (Fig. 6d and 7d), all indicating that AMPK inactivation is the downstream signaling of LTB4 effects, as previously reported^22^. These results provide conclusive evidence that AMPK activation is required for the protective effects of zileuton in C2C12 myotubes.

**Figure 8:**
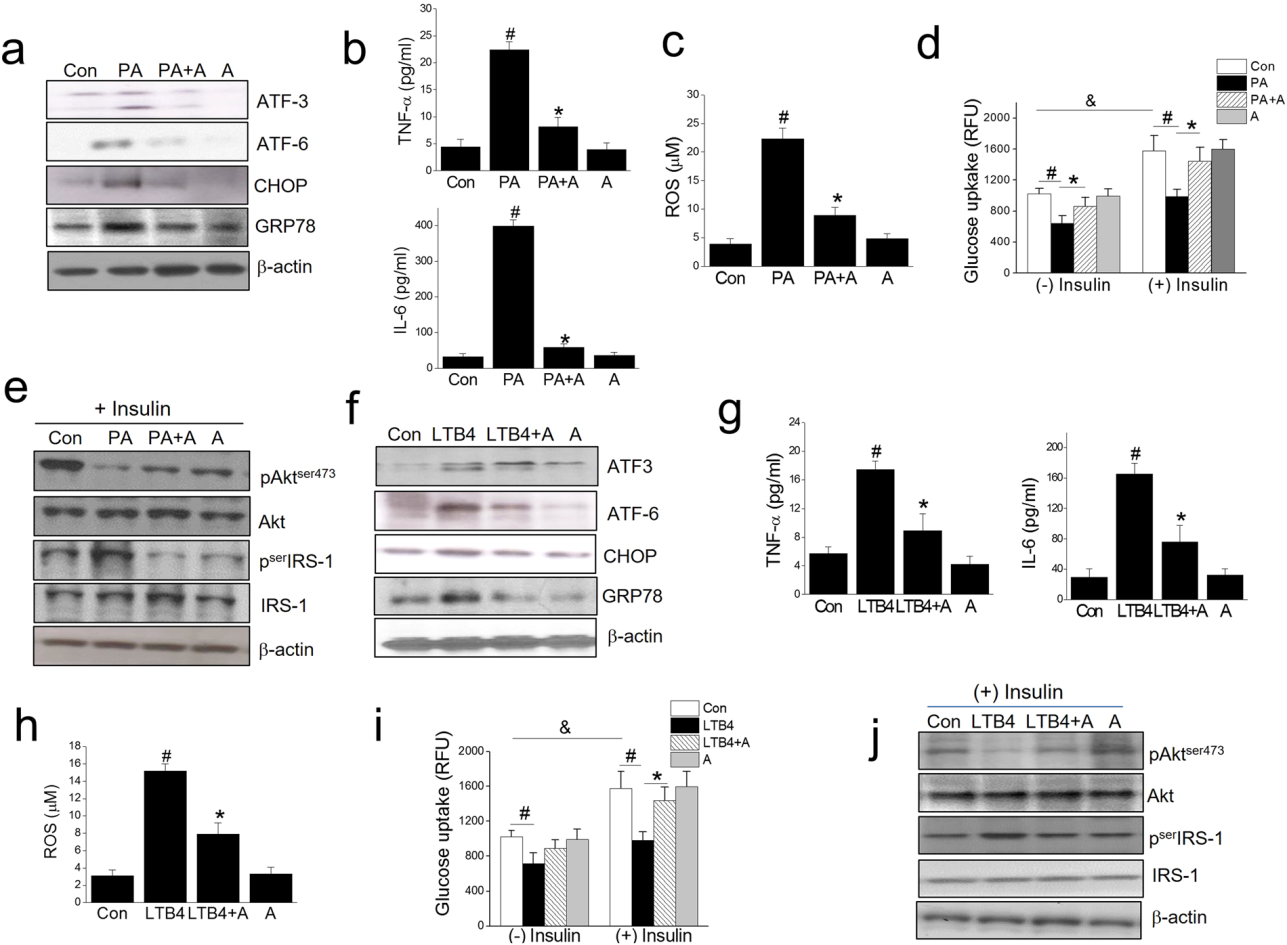
The effect of an AMPK activator, A769662, on the PA and LTB4 actions. Differentiated C2C12 myotubes were exposed to A769662 (80 μM) for 1 h and then treated with either PA (750 μM) or LTB4 (100 ng/ml) in the presence of A769662 for 12 h (qPCR) or 24 h (ELISA). Levels of ER stress markers were determined by western blotting (**a** and **f**). Levels of pro-inflammatory cytokines were determined by ELISA (**b** and **g**). ROS production was assessed using AmplexRed (**c** and **h**). After 24 h of PA treatment, insulin (1 μg/ml) was added for 30 min, and then glucose uptake was determined by 2-NBDG (**d** and **i**). Akt and serIRS-1 phosphorylation was assessed by western blotting (**e** and **j**). The results of the western blotting are shown as the representative of three independent experiments (**a**,**e**,**f**,**j**), and other results are expressed as the means ± SDs of three experiments performed in triplicate. ^#^ *P* < 0.05 *vs*. non-treated controls, *P < 0.05 *vs*. PA or LTB4 alone, ^&^ *P* < 0.05 *vs*. (−) insulin.

Next, we examined whether AMPK activation could prevent PA or LTB4-induced ER stress, inflammation and insulin resistance to provide further evidence that AMPK inactivation by LTB4 is a critical player in PA action. As shown in Fig. 8a–e, A769662 (80 μM), an AMPK activator, reduced PA-induced ER stress, ROS, proinflammatory cytokines, in parallel with enhanced insulin signaling and glucose uptake. Similar effects of A769662 were observed when LTB4 was treated in place of PA on C2C12 myotube (Fig. 8f–j).

To elucidate the mechanisms through which zileuton activates AMPK, the involvement of Ca^2+^/calmodulin dependent protein kinase pathway was examined. Intracellular Ca^2+^ was unaltered by zileuton, and BATA-AM (a Ca^2+^ chelator) had no effect on zileuton-induced AMPK activation, indicating that Ca^2+^/calmodulin dependent protein kinase is not responsible for zileuton-induced AMPK activation. In addition, no difference in the association between AMPK and LKB1 was observed by zileuton, which suggests that LKB1 pathway is not likely involved in zileuton-induced AMPK activation (Supplementary Fig. 2). On the other hand, AMPK phosphorylation by zileuton was reduced by PP2 (a c-src kinase inhibitor), which was also shown in previous reports that c-src mediates AMPK activation^23, 24^.

### ***In vivo*** effects of zileuton in *ob/ob* mice

**Figure 9:**
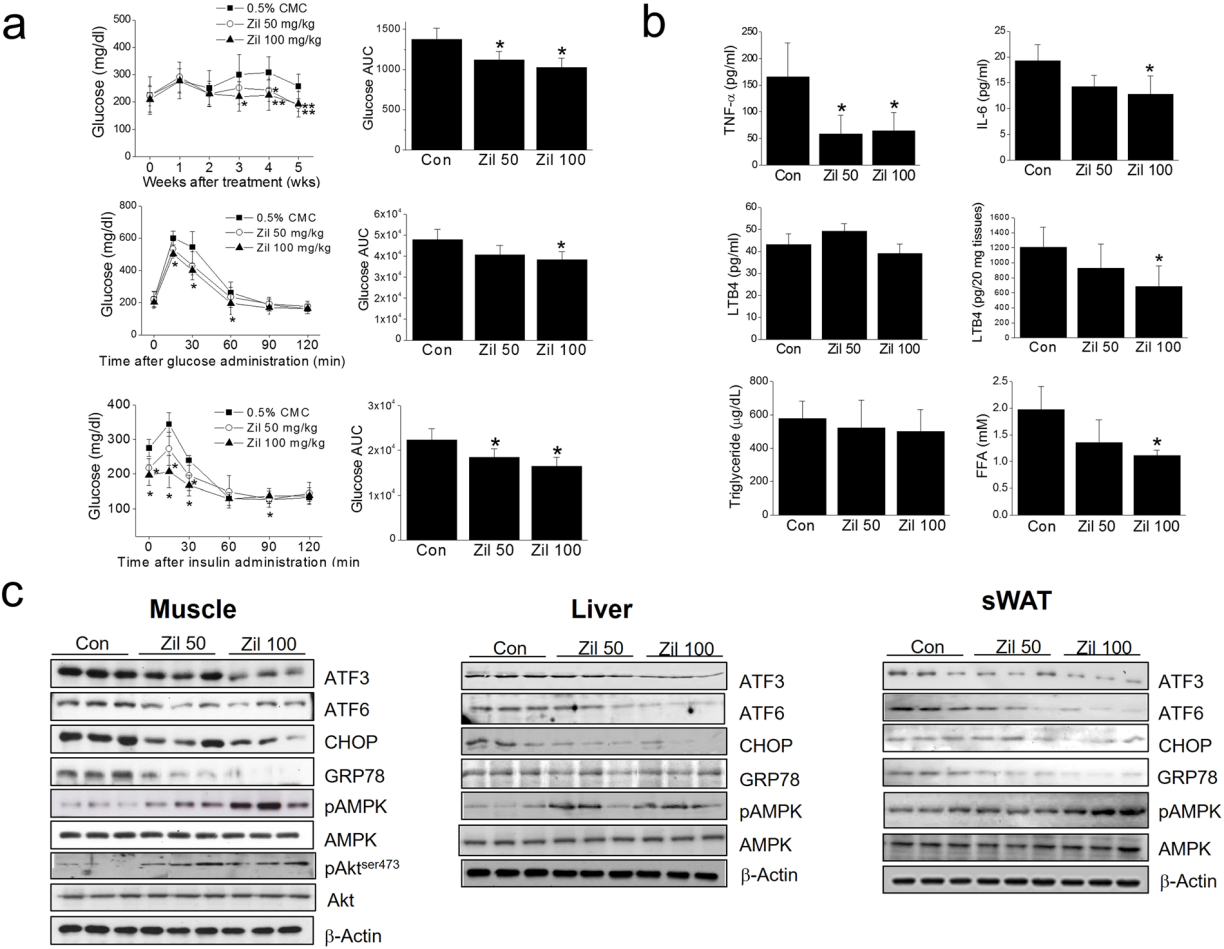
*In vivo* effects of zileuton in *ob/ob* mice. Zileuton (50 or 100 mg/kg, n = 10 each group) was orally administrated to *ob/ob* mice once daily for 5 weeks; plasma glucose concentrations were determined weekly from tail veins (**a**, top). At the end of the experiment, oral glucose tolerance (**a**, middle) and insulin tolerance testings (**a**, bottom) were conducted, and plasma glucose AUC was calculated using OriginPro 6.1 software. The plasma levels of pro-inflammatory cytokines, LTB4, FFA and triglyceride were measured by ELISA kits (**b**). Muscle LTB4 was determined by ELISA kits (**b**). The expression levels of ER stress markers, and AMPK and Akt phosphorylation levels in skeletal muscles, liver and adipose tissues were determined by western blotting (**c**). *P < 0.05, **P < 0.01 *vs*. non-treated controls.

To explore the effects of zileuton *in vivo*, *ob/ob* mice, an animal model of T2DM, were orally administered zileuton (50 or 100 mg/kg) once daily for 5 weeks. Zileuton lowered blood glucose levels as compared with vehicle (0.5% CMC) treated controls (Fig. 9a, top), and improved glucose intolerance as determined by OGTT and ITT (Fig. 9a, middle and bottom). Furthermore, plasma levels of TNF-α, IL-6 and FFA were reduced by zileuton (Fig. 9b). Surprisingly, plasma levels of LTB4 were not altered by zileuton, but muscle LTB4 was decreased in zileuton-treated group (Fig. 9b). In-line with our *in vitro* findings, in zileuton-treated mice, the expressions of ER stress markers were reduced with increased AMPK and Akt phosphorylation in skeletal muscle (Fig. 9c). Separately, we also determined the effects of zileuton in liver and adipose tissues, and similar protective effects of zileuton were occurred as expected from systemic exposure of zileuton (Fig. 9c). These results suggest that zileuton ameliorates insulin resistance *in vivo*, and that direct activation of skeletal AMPK at least partly contributes to its therapeutic effects.

## Discussion

Leukotrienes are potent proinflammatory mediators, and the LTB4-BLT1 axis plays an important role in obesity-driven inflammation and insulin resistance^16^. This study expands knowledge regarding the role of LTB4 in skeletal insulin resistance, and 5-LO suppression could ameliorate PA-induced insulin resistance in murine skeletal muscle. In addition, we report that AMPK activation is involved in the protective effects of 5-LO suppression against PA-induced insulin resistance.

During recent years, studies on the 5-LO pathway and its leukotriene metabolites have increased understanding of the pathophysiological aspects of metabolic diseases, including T2DM^16, 18^. Cysteinyl leukotrienes (LTC4, D4, and E4) are known to stimulate contraction of bronchial smooth muscles, whereas LTB4 is a potent chemoattractant, and stimulates adipose tissue infiltration by macrophages and immune cells^16, 25^. LTB4 also stimulates the productions of other proinflammatory cytokines, and thus, causes low grade chronic inflammatory states^17^. Leukotriene levels are elevated in obese mice and humans^15^, and linkage analyses have recently shown the 5-LO gene influences adipose fat accumulation^26^. In addition, high fat diet animal models have shown that 5-LO or BLT1 deficiency improves insulin resistance, and that this increased insulin sensitivity is probably caused by reduced macrophage and proinflammatory T cell numbers in adipose tissue^19^. On the other hand, LTB4 inhibition reduced lipolysis in adipose tissue, and thus, plasma levels of FFA in diet-induced obese mice^22^.

The pathological roles of the 5-LO pathway in hepatic insulin resistance and inflammatory disease have been recently described. BLT1 deletion was found to decrease hepatic triglyceride accumulation and inflammation, and have beneficial effects on hepatosteatosis and nonalcoholic fatty liver disease^18, 27, 28^. The beneficial effects of BLT1 deletion in liver may have been due to indirect effects, because adipose LTB4 can be delivered to liver through an extensive vascular network. On the other hand, recent reports suggest that increased hepatic LTB4 directly affects hepatic insulin sensitivity, and thus in high fat diet-induced mice and in leptin deficient *ob/ob* mice, the beneficial effects of zileuton on insulin resistance appeared to be due to the combined actions of adipose and hepatic tissues^29^.

Recent studies have demonstrated exogenous LTB4 (100 nM) in L6 myotubes evokes insulin resistance via a Gαi-coupled BLT1 signaling pathway involving serIRS-1 phosphorylation and a subsequent decrease in Akt phosphorylation^16^. In the present study, C2C12 murine myotubes were found to express 5-LO and BLT1 receptor, thus indicating the constitutive existence of a functional LTB4-BLT1 pathway, consistent with previous reports^20, 21^. Correspondingly, PA induced LTB4 production with increased ER stress and oxidative stress and impaired insulin signaling, suggesting that LTB4 may act in an autocrine fashion to induce skeletal insulin resistance. Conversely, inhibition of 5-LO pathway (*i*.*e*. zileuton, 5-LO siRNA and BLT1 receptor antagonist) prevented impaired insulin signaling by PA as evidenced by the inhibitions of PA-induced serIRS phosphorylation and reduced Akt phosphorylation, which is consistent with previously reported results^16^. In the present study, the level of PA-induced LTB4 secretion into culture medium was low (~150 pM), but it is plausible chronic exposure of low LTB4 still could induce insulin resistance in local environment. In fact, LTB4 concentrations in the adipose tissues of lean and obese mice have been reported to be in sub pg/mg of tissue range^15^. Although our results suggest that local LTB4 elicits direct effects on myotubes, the possibility that LTB4 produced locally in skeletal muscle induces macrophage infiltration into skeletal muscle *in vivo*, and thus, leads to inflammation and insulin resistance cannot be excluded, since in a previous study, macrophage depletion and BLT1 inhibition improved glucose intolerance in an additive manner^19^.

AMPK regulation is of considerable interest in T2DM, because accumulating data indicates AMPK activation protects against T2DM and insulin resistance^30, 31^. Importantly, AMPK activation has been shown to suppress ROS production and inflammation via NF-*k*B inhibition in T2DM patients^32^. Consistent with such reports, our findings indicate zileuton significantly induces AMPK phosphorylation and subsequently ameliorates PA-induced ER stress and insulin resistance. We found AMPK siRNA or compound C (an AMPK inhibitor) abolished the protective effects of zileuton on ER stress marker induction, ROS production, pro-inflammatory cytokine secretion, and AMPK suppression by AMPK siRNA prevented insulin-stimulated Akt and serIRS-1 phosphorylation by zileuton. Furthermore, PA or LTB4-induced lipotoxicity was lessened in the presence of an AMPK activator. These results strongly support the notion that AMPK critically mediates the activity of zileuton. It remains unclear how zileuton activates AMPK, and further in depth studies are required. On the other hand, src activation was detected by zileuton treatment, and PP2 (a src inhibitor) reduced zileuton-induced AMPK activation, suggesting that src activation may lead to AMPK activation.

The expression of 5-LO activating protein (FLAP; a facilitator of the interaction between arachidonic acid and 5-LO) has been reported to be elevated in obese mice^22, 33^. Furthermore, in accord with our results, FLAP inhibition by Bay-X-1005 and LTB4 receptor antagonist (U75302) have been reported to induce AMPK phosphorylation in diet-induced obese mice, which suggests AMPK activation importantly underlies the protective effects exerted by 5-LO inhibition^22, 34^. These findings are in agreement with our observations that 5-LO knockdown induces AMPK phosphorylation during PA stimulation. On the other hand, AMPK siRNA had little effect on the suppressive effects of zileuton on LTB4 production, reinforcing that AMPK activation is a downstream signaling of LTB4 inhibition by zileuton. Separately, previous studies demonstrate that lipid metabolites such as ceramide and diacylglycerol are involved in PA action^35, 36^. In line with this, exogeneous LTB4 induced both ceramide and diacylglycerol in primary hepatocytes^16^. Based on these observations, it may be possible that 5-LO inhibition results in the reduction of lipid metabolites, restoring PA-induced impairment of insulin sensitivity in myotube.

Studies suggest ER is highly sensitive to redox status^7, 8^, and accumulations of misfolded proteins in ER lumen are known to produce ROS^37^ followed by c-Jun N-terminal kinase (JNK) activation and increased serIRS-1 phosphorylation, and insulin resistance^38, 39^. Thus, excessive ROS generation appears to cause impairments of insulin signaling and glucose disposal in skeletal muscle^40, 41^, and it is for this reason we evaluated the involvement of ROS in the effects of LTB4. Based on our study, ROS increase by PA might induce 5-LO and BLT1 receptor expression, reinforcing further PA-induced lipotoxicity. Reversely, we observed direct inhibition of 5-LO by zileuton reduced PA-induced ROS production in C2C12 myotubes and restored insulin sensitivity.

Prompted by our *in vitro* observations, we investigated the *in vivo* effects of zileuton in *ob/ob* mice (a murine model of T2DM). In line with previous reports on zileuton in high fat diet-induced obese mice^15^, daily zileuton (50 or 100 mg/kg) for 5 weeks reduced glucose intolerance, levels of ER stress markers, and enhanced AMPK phosphorylation in skeletal muscle. Although plasma levels of LTB4 was relatively low (approximately 40 pg/ml) and was little affected, zileuton significantly reduced muscle LTB4 to 56% of vehicle group. These results suggest zileuton acts directly on skeletal muscle cells, and that this results in reduced local LTB4 levels albeit not systemically and the attenuation of insulin resistance. Besides, adipose and liver LTB4 levels were also reduced by zileuton, in association with reduced ER stress marker expression, suggesting that systemic exposure of zileuton may produce insulin sensitizing effect. Importantly, AMPK activation resulting from LTB4 reduction appears to be a key player in 5-LO inhibition-induced insulin sensitization in skeletal muscle. Taken together, our findings demonstrate that AMPK activation induced by skeletal 5-LO inhibition ameliorates murine insulin resistance, and highlight the possible therapeutic applications of 5-LO inhibition for the treatment of obesity-induced metabolic diseases like T2DM.

## Methods

### Chemicals

Mouse C2C12 myoblasts were obtained from the American Type Culture Collection (ATCC, Rockville, MD). Dulbecco’s modified Eagle’s medium (DMEM), fetal bovine serum (FBS), Dulbecco’s phosphate buffered saline (D-PBS), horse serum, penicillin, and streptomycin were from GIBCO (Grand Island, NY). Zileuton and LTB4 were from Tocris (Bristol, UK), and AmplexRed from Invitrogen (Waltham, MA). A769662 was obtained from LC laboratories (Woburn, MA). Antibodies were obtained as follows: GRP78, ATF3, CHOP, Akt and pAkt (Ser473) (Thr307) from Santa Cruz Biotechnology (Santa Cruz, CA); AMPK, pAMPK, pACC (Ser79) and ACC from Cell Signaling Technology (Danvers, MA); pIRS-1 (Ser307) and IRS-1 from Bioworld (Minneapolis, MN); 5-LO from Novusbio (Littleton, CO) and β-actin from Sigma-Aldrich (St. Louis, MO). 5-LO siRNA (sc-29597), AMPK1/2 siRNA (sc-45313) and control siRNA (sc-37007) were from Santa Cruz Biotechnology (Santa Cruz, CA). Oligonucleotide primers were from Bioneer Co. Ltd. (Daejeon, Korea). LTB4 kits were from Enzo Life Sciences (Seoul, Korea) and MyBioSource, Inc. (San Diego, CA). CysLTs kit and U75032 were from Cayman chemicals (Ann Arbor, MI). IL-6 and TNF-α ELISA kits were from BD Biosciences (San Diego, CA). All other reagents were from Sigma Chemicals (St. Louis, MO) unless indicated otherwise.

### Animals

Male *ob/ob* mice (5 weeks old) were purchased from the Korean Research Institute of Bioscience and Biotechnology (Ochang, Korea). Animals were housed under standard animal care conditions (20 ± 2 °C and humidity 40–60% under a 12 h light/dark cycle). After acclimatization for 1 week, mice were orally administered vehicle (0.5% CMC, n = 10) or zileuton (50 or 100 mg/kg, n = 10 each dose) once daily for 5 weeks. Body weights and food intakes were measured weekly at the same time every week (between 10:00 and 11:00 AM) during the 5-week experimental period. Blood glucose concentrations in tail veins were determined in a randomly fed state using an Allmedicus Gluco Dr. Plus glucometer (Seoul). All animal procedures were performed in accordance with the Guide for the Care and Use of Laboratory Animals published by the US National Institute of Health (NIH Publication No. 85-23, revised 2011) and approved by the Animal Care and Use Committee of Gachon University.

### Cell culture and differentiation

C2C12 myoblasts were maintained in DMEM supplemented with 10% heat-inactivated FBS, penicillin (100 units/ml), and streptomycin sulfate (100 µg/ml) in a humidified 5% CO_2_ atmosphere at 37 °C. When cells had achieved 100% confluence, myoblast to myotube differentiation was induced by replacing media with DMEM containing 2% horse serum. After a 4-day differentiation period, myotubes were used for the experiments. All experiments in this study were conducted on differentiated myotubes unless otherwise indicated.

### PA solution preparation

BSA-bound PA was prepared as previously described^42^. Briefly, PA was completely dissolved in 100% ethanol and then diluted in DMEM containing 2% fatty acid-free BSA. The control treatment was prepared by adding the same amount of ethanol to BSA-DMEM solution. All solutions were filtered, aliquoted, and stored at −20 °C prior to use.

### Cell viability assay

C2C12 myotubes in 12-well plates were incubated with various concentrations of zileuton (1–50 μM) for 1 h and then exposed to PA (750 μM) for 24 h. At the end of treatment, cells were cultured with MTT at a final concentration of 0.5 mg/mL for 4 h. The purple formazan crystals produced were dissolved using DMSO, and absorbances were measured using a spectrophotometer at 570 nm (Perkin Elmer VictorX4, Waltham, MA). Cell viabilities (%) were calculated by comparing the absorbances of samples and the control. LDH assay was carried out using the Cytotoxicity Detection kitPLUS (Roche Diagnostics Gmbh) according to the manufacturer’s instructions. Briefly, differentiated C2C12 cells in the presence of various PA concentrations were incubated for 24 h. Culture supernatant (50 μl) from each well was transferred to a 96-well plate and 100 μl reaction mixture solution was added to each well. Following incubation at room temperature in the dark for 30 min, 50 μl of stop solution was added to each well to terminate the reaction. Absorbance was measured using a microplate reader at 490 nm. The percentage of cytotoxicity is determined by calculating the optical density at 490 nm (OD_490nm_) and subtracting from the absorbance value obtained in the background control.

### Western blot analysis

Cells or excised tissues were lysed in PRO-PREP™ protein extraction solution (Intron Biotechnology, Korea) and centrifuged at 14000 g for 15 min. Aliquots of lysates (30 μg) were subjected to western blot analysis as previously reported^43^. All protein levels were normalized with respect to β-actin or total protein levels.

### RNA preparation and real-time PCR (qPCR)

Total RNA was isolated from C2C12 myotubes and from mouse muscle using Trizol (Invitrogen, Carlsbad, CA), and complementary deoxyribonucleic acid (cDNA) was synthesized using ReverTra Ace qPCR RT master mix (Toyobo, Japan). Real-time PCR was performed in triplicate using a SYBR Green Master mix (Toyobo, Japan) and a Roche Light cycler 2.0 (Roche Bio Inc., Switzerland). The expression levels of the target genes were calculated versus endogenous GAPDH. The sequences of the mouse primers used are listed below; GRP78: 5′-CTGGACTGAATGTCATGAGGATCA-3′ (F) and 5′-CTC TTA TCC AGG CCA TAT GCA ATA G-3′ (R), ATF3: 5′-AAC TGG CTT CCT GTG CAC TT-3′ (F) and 5′-TGA GGC CAG CTA GGT CAT CT-3′ (R), ATF6: 5′-TCG CCT TTT AGT CCG GTT CTT-3′ (F) and 5′-GGC TCC ATA GGT CTG ACT CC-3′ (R), CHOP: 5′-CCA CCA CAC CTG AAA GCA GAA-3′ (F) and 5′-GGT GCC CCC AAT TTC ATC T-3′ (R), IL-6: 5′-TCT AAT TCA CTT CAA CCA AGA GG-3′ (F) and 5′-TGG TCC TTA GCC ACT CCT TC-3′ (R), TNF-α: 5′-CAT GCA CCA CCA TCA AGG ACT-3′ (F) and 5′-GAG GCA ACC TGA CCA CTC TC-3′ (R), 5-LO: 5′-ATG TTG GCA TCT AGG TGC AGT GTG-3′ (F) and 5′-ATC ATG GCT TCC TTC ACT GGC TTC-3′ (R), BLT1: 5′-TCC CTT TTT CCT CCA CTT TC-3′ (F) and 5′-GAA AAG ACA CCA CCC AGA TG-3′ (R), BLT2: 5′-ACA GCC TTG GCT TTC TTC AG-3′ (F) and 5′-TGC CCC ATT ACT TTC AGC TT-3′ (R), CysLT1: 5′-TTG AGC CTC CAC AGA ACA ATC-3′ (F) and 5′-TTC CTA CGA CTT GGC ATG TTT T-3′ (R), CysLT2: 5′-TGG GAT AAA GAT TCA TGT GGG GA-3′ (F) and 5′-AGC CCT TAA TCG AGC TTT GAA A-3′ (R) and GAPDH: 5′-CTC AAC TAC ATG GTC TAC ATG TTC CA-3′ (F) and 5′-CCA TTC TCG GCC TTG ACT GT-3′ (R).

### AMPK and 5-LO knockdown by siRNA transfection

AMPK or 5-LO siRNA (both 20 nM; Santa Cruz Biotechnology) or negative control siRNA were transfected into differentiated C2C12 cells using Lipofectamine^TM^ RNAiMAX (Invitrogen, Madison, WI) in Opti-MEM medium according to the manufacturer’s instructions. Cells were then incubated for 24 h and media were replaced with differentiation medium. After 48 h of incubation, the C2C12 myotubes were exposed to PA (750 μM) for 24 h in the presence or absence of the indicated concentrations of zileuton. Efficiencies of AMPK and 5-LO knockdowns were determined by western blotting against AMPK and 5-LO antibodies.

### 2-NBDG glucose uptake assay

Glucose uptake was measured by adding the fluorescent D-glucose analogue 2-NBDG (2-(N-(7-nitrobenz-2-oxa-1,3-diazol-4-yl)amino)-2-deoxyglucose) (Cayman Chemicals, Arbor, MI) as previously described^44^. Briefly, differentiated C2C12 myotubes in 96 well black plates (BD Bioscience, Bedford, MA) were incubated with serum-free DMEM containing 2-NBDG (10 μM) in the presence or absence of insulin (1 μg/ml) for 30 min. Medium was removed and cells were washed with PBS twice and then incubated with lysis buffer (0.1 M potassium phosphate, 1% Triton X-100, pH 10) for 10 min with shaking. Dimethyl sulfoxide (DMSO) was then added with shaking and allowed to react for 20 min. Amounts of 2-NBDG taken up by cells were determined by measuring fluorescence intensities at λ_ex_ 466 nm and λ_em_ 550 nm using a microplate reader (Perkin Elmer VictorX4, Waltham, MA).

### Detection of hydrogen peroxide using Amplex Red

ROS accumulations in C2C12 myotubes were measured using an Amplex Red Hydrogen Peroxide assay Kit (Invitrogen, Waltham, MA) according to the manufacturer’s instructions. C2C12 myotubes were pre-treated with the indicated concentrations of zileuton (1–50 μM) for 1 h, and treated with PA (750 μM) for 24 h. Cells were then re-suspended with 100 μl of Krebs Ringer phosphate buffer containing 5.5 mM glucose. Supernatants were harvested and assayed (in duplicate) in 96-well fluorescent assay plates (Thermo Fisher Scientific) containing 50 μl/well of Amplex Red solution with 0.2 U HRP. Fluorescence was recorded using a fluorometer (λ_ex_ 540 nm; λ_em_ 590 nm). H_2_O_2_ concentrations were determined using a standard curve.

### Immunoprecipitation of LKB1

Cells were lysed in the RIPA buffer, and then, protein (0.5 mg) from cell lysate was immunoprecipitated using rabbit anti-AMPK antibody (1:100) with prewashed protein G Plus-Agarose overnight. Protein complex was centrifuged at 3,000 rpm for 3 min at 4 °C, and the pellets were washed three times with 10 volumes of ice-cold RIPA buffer. The final pellet was re-suspended with SDS sample buffer, heated for 5 min at 95 °C and separated by SDS-PAGE. After separation, proteins were transferred to PVDF membrane and immunoblotted with LKB antibody.

### Intracellular calcium measuring using FACS

Intracellular calcium concentrations were measured with a fluorescent calcium indicator Fura Red AM and fluo-3 AM (Invitrogen, Burlington, ON). Differentiated C2C12 myotube (0.5 × 10^6^ cells) were incubated with PA in the presence or absence of zileuton for 6 h. Cells were washed twice with PBS and incubated for 30 min with 4 μg/ml fluo-3 AM and 10 μg/ml Fura Red AM in HBSS containing 1 mM Ca^2+^, 1 mM Mg^2+^ and 1% FBS. After incubation, cells were washed twice with PBS and re-suspended the final pellets with 1 ml HBSS containing 1 mM Ca^2+^, 1 mM Mg^2+^ and 1% FBS. Intracellular Ca^2+^ was analyzed by flow cytometry (FACSCanto II, BD bioscience, San Jose, CA).

### ELISA for IL-6, TNF-α, LTB4 and CysLTs

Secreted TNF-α and IL-6 levels were measured using commercially available ELISA kits according to the manufacturer’s instructions. The levels of LTB4 in tissue and supernatants were measured by ELISA according to manufacturer’s instructions. Briefly, supernatant (100 μl) were incubated with polyclonal LTB4 binding wells for 2 h. After stopping of enzymatic reaction, optical density was measured at 405 nm. To determine CysLTs in supernatant, supernatant (50 μl) was incubated with CysLT AceE tracer and CysLT monoclonal antibody in goat anti-mouse IgG pre-coated 96 well plate, and then incubated overnight at 4 °C. After washing, Ellman’s reagent was added into each well and read the absorbance at 405 nm.

### Serum triglyceride and free fatty acid measurements

Serum levels of triglycerides (TGs) and free fatty acids (FFAs) were measured using assay kits (Cell Biolabs, Inc., San Diego, CA) as described by manufacturers’ protocols. Absorbance was then measured at 540–570 nm using a microplate reader.

### Oral glucose tolerance testing (OGTT) and insulin tolerance testing (ITT)

OGTT and ITT were conducted after 5 weeks of zileuton treatment. For OGTT, mice were fasted for 16 h (from 5:00 PM to next day 9:00 AM) prior to testing and then given an oral injection of D-glucose (2 g/kg). Blood glucose was measured using tail blood at different time points. Mice were allowed to recover for 3 days after OGTT. For ITT, mice were starved for 4 h (from 9:00 AM to 1:00 PM) and 0.75 IU/kg insulin (Sigma Chemical, St. Louis, MO) was administered intraperitoneally. Blood glucose levels were measured at indicated time points using an Allmedicus Gluco Dr. Plus glucometer (Seoul, Korea). Areas under the curve (AUCs) for glucose were calculated for the OGTT and ITT using OriginPro 6.1 software (Origin, Northampton, MA).

### Tissue preparation and analysis

All mice were sacrificed on day after ITT, and tissues including muscle were removed, rinsed with ice cold phosphate buffered saline (PBS) to remove excess blood thoroughly, and weighed before homogenization. For RNA and protein extraction, tissues were minced to small pieces and homogenized them in PBS (usually10 mg/100 μl) with a glass homogenizer on ice. The resulting suspension was subjected to ultrasonication or to two freeze-thaw cycles to further break the cell membranes. Then, the homogenates were centrifugated for 15 minutes at 1500× g (or 5000 rpm).

### Statistical analysis

Significances of differences versus respective controls were determined using the Student’s *t*-test for paired experiments or two-way ANOVA. Results are presented as the means ± SDs of three separate experiments, and *p* values of <0.05 were considered statistically significant.

## Electronic supplementary material

Supplementary information
