# Supplementary material for: 5-LO inhibition ameliorates palmitic acid-induced ER stress, oxidative stress and insulin resistance via AMPK activation in murine myotubes
Source: Sci Rep. 2017 Jul 10;7:5025. doi: 10.1038/s41598-017-05346-5 (PMC5504062; doi:10.1038/s41598-017-05346-5)
Supplement: Supplementary file 1 — Supplementary information [file 41598_2017_5346_MOESM1_ESM.pdf]

5-LO inhibition ameliorates palmitic acid-induced ER stress, oxidative stress and insulin resistance via AMPK activation in murine myotubes

Hyun Jeong Kwak[a], Hye-Eun Choi[a], Hyae Gyeong Cheon[a,b*]

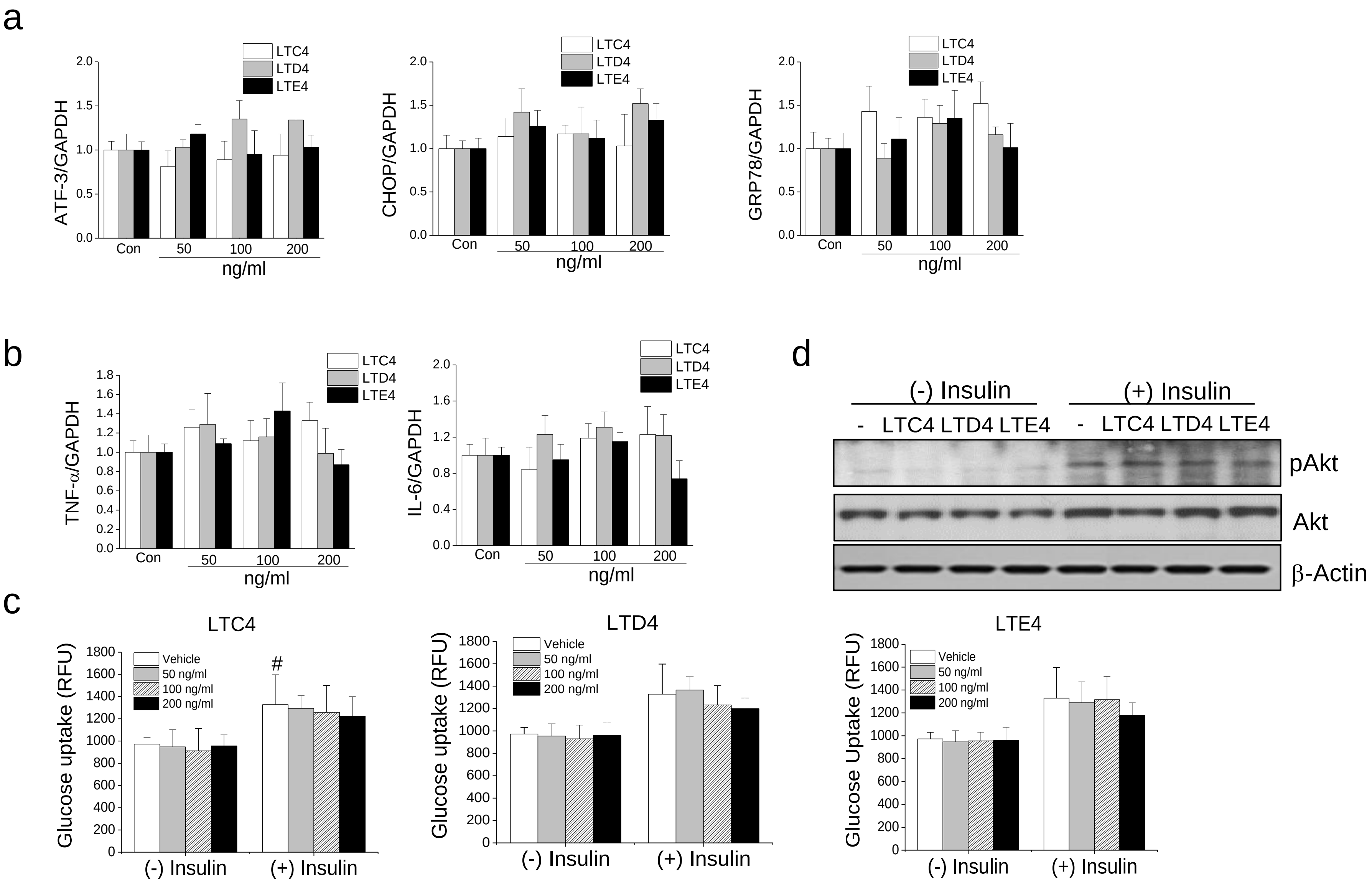

Supplementary Figure 1. Effects of CysLTs on ER stress marker, proinflammatory cytokines, Akt phosphorylation and glucose uptake. C2C12 myotubes were exposed to each CysLT such as LTC4, LTD4 or LTE4 (0-200 ng/ml) for 12 h. The expression levels of ER stress markers and proinflammatory cytokines were then determined by qPCR (a and b). After 24 h of CysLTs treatment, insulin (1 mg/ml) was added for 30 min, and then glucose uptake was determined by 2-NBDG (c). Akt phosphorylation was assessed by western blotting (d). The results of the western blotting are shown as the representative of three independent experiments (d), and other results are expressed as the means ± SDs of three experiments performed in triplicate. #*P*<0.05 *vs.* non-treated controls.

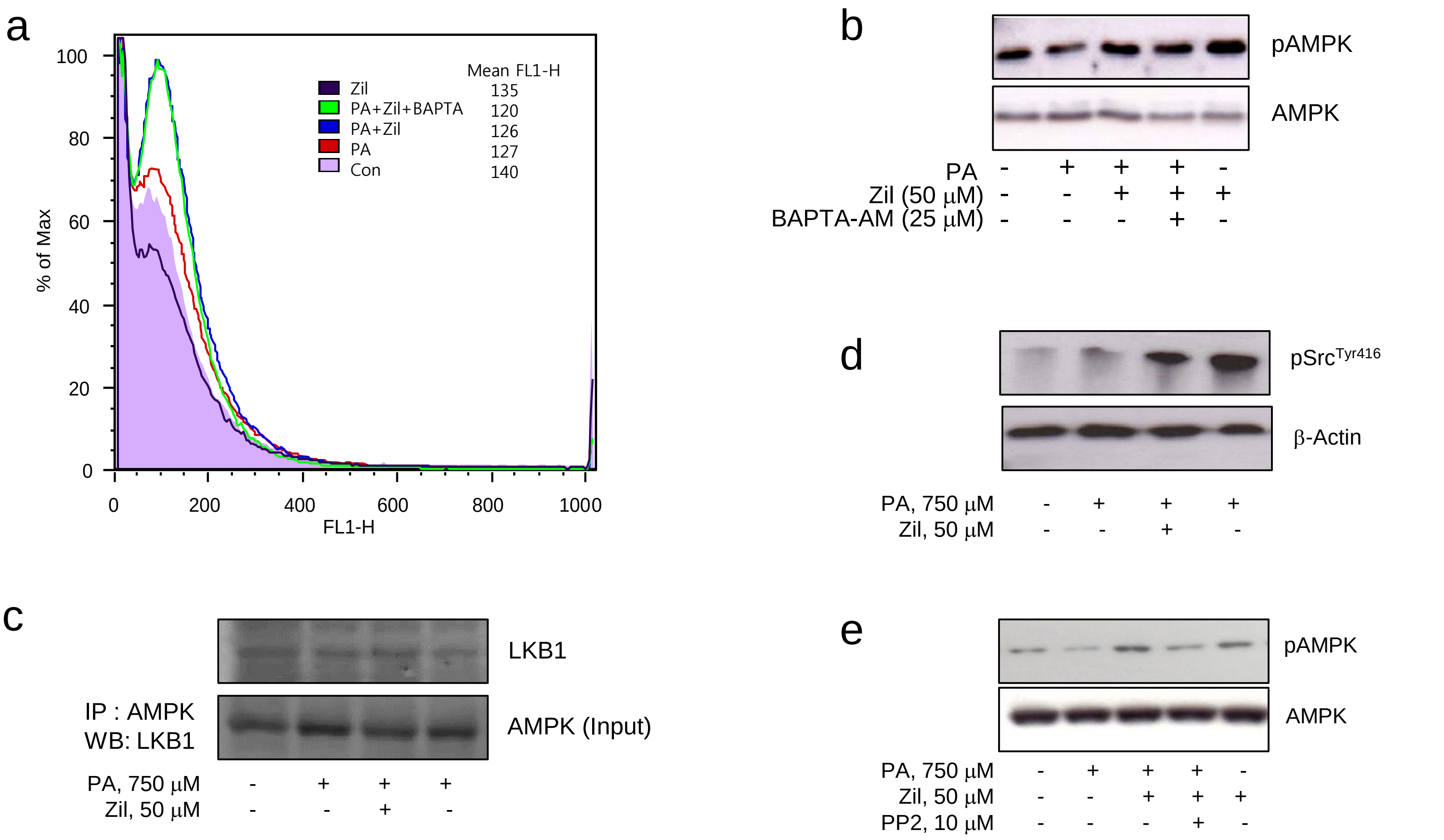

Supplementary Figure 2. Zileuton-induced AMPK phosphorylation is mediated through Src activation independent of Ca^{2+} -and LKB1-mediated pathway. C2C12 myotubes were exposed to zileuton (50 μM) for 1 h with or without BAPTA-AM (25 μM) or PP2 (10 μM) followed by PA (750 μM) treatment for 6 h (a) or 24 h (b-e). (a) Intracellular Ca^{2+} release from cells loaded with the Ca^{2+} indicator Fluo-4 AM and Fura red. (b) AMPK phosphorylation was detected in the presence or absence of BAPTA-AM, a Ca chelator, by Western blot analysis. (c) Cell extracts were immunoprecipitated with anti-AMPK antibody and then immunoblotted with anti-LKB1. Immunoblot with AMPK used as input control. (d) The Thr^{Tyr416} phosphorylation of Src was detected by Western blot analysis. (e) AMPK phosphorylation was detected in the presence or absence of PP2, a Src kinase inhibitor.

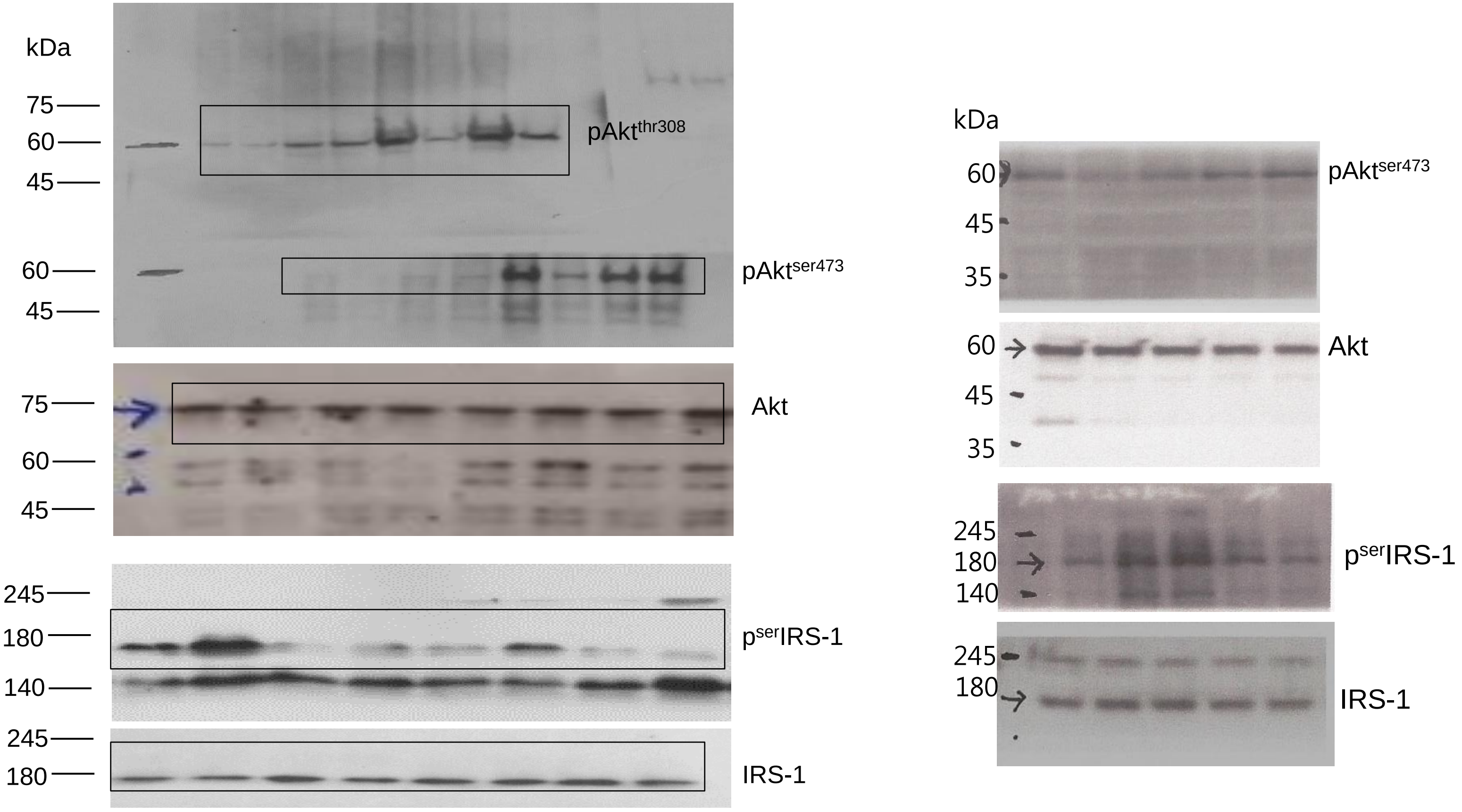

Supplementary Figure 3. Uncropped scans of western blot displayed in Fig. 2b and Fig. 4d

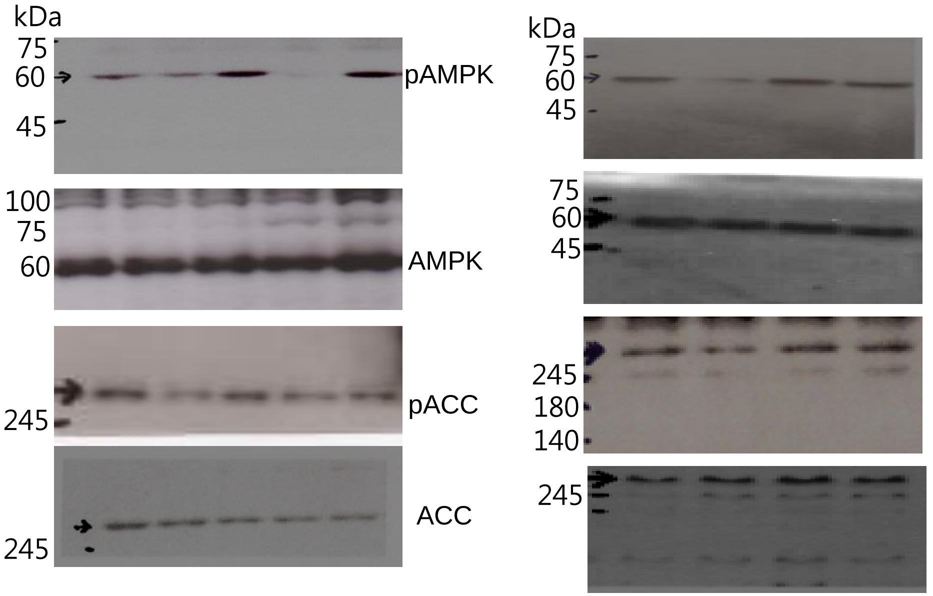

Supplementary Figure 4. Uncropped scans of western blot displayed in Fig. 6a and b

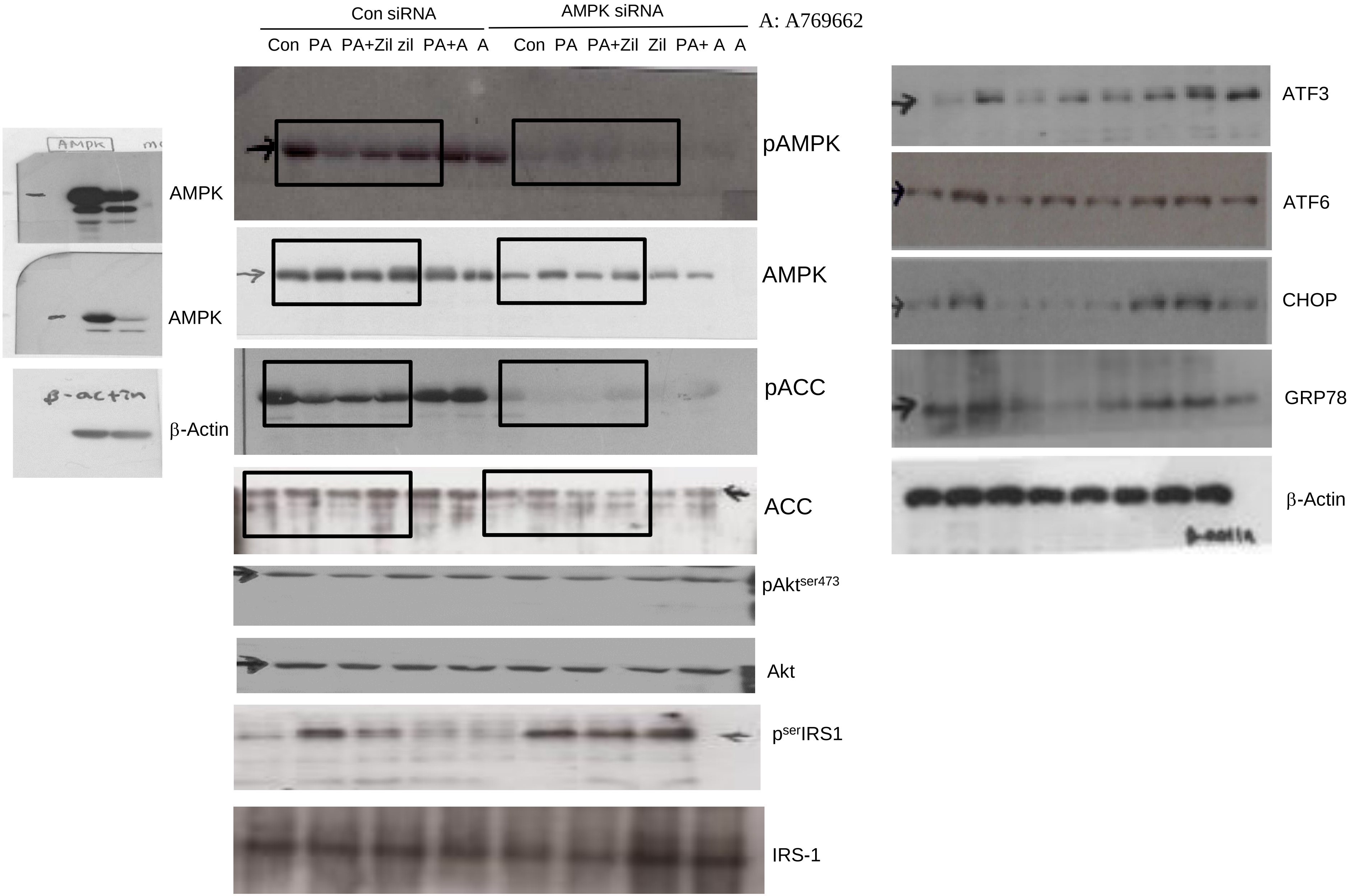

Supplementary Figure 5. Uncropped scans of western blot displayed in Fig. 7a, b and c

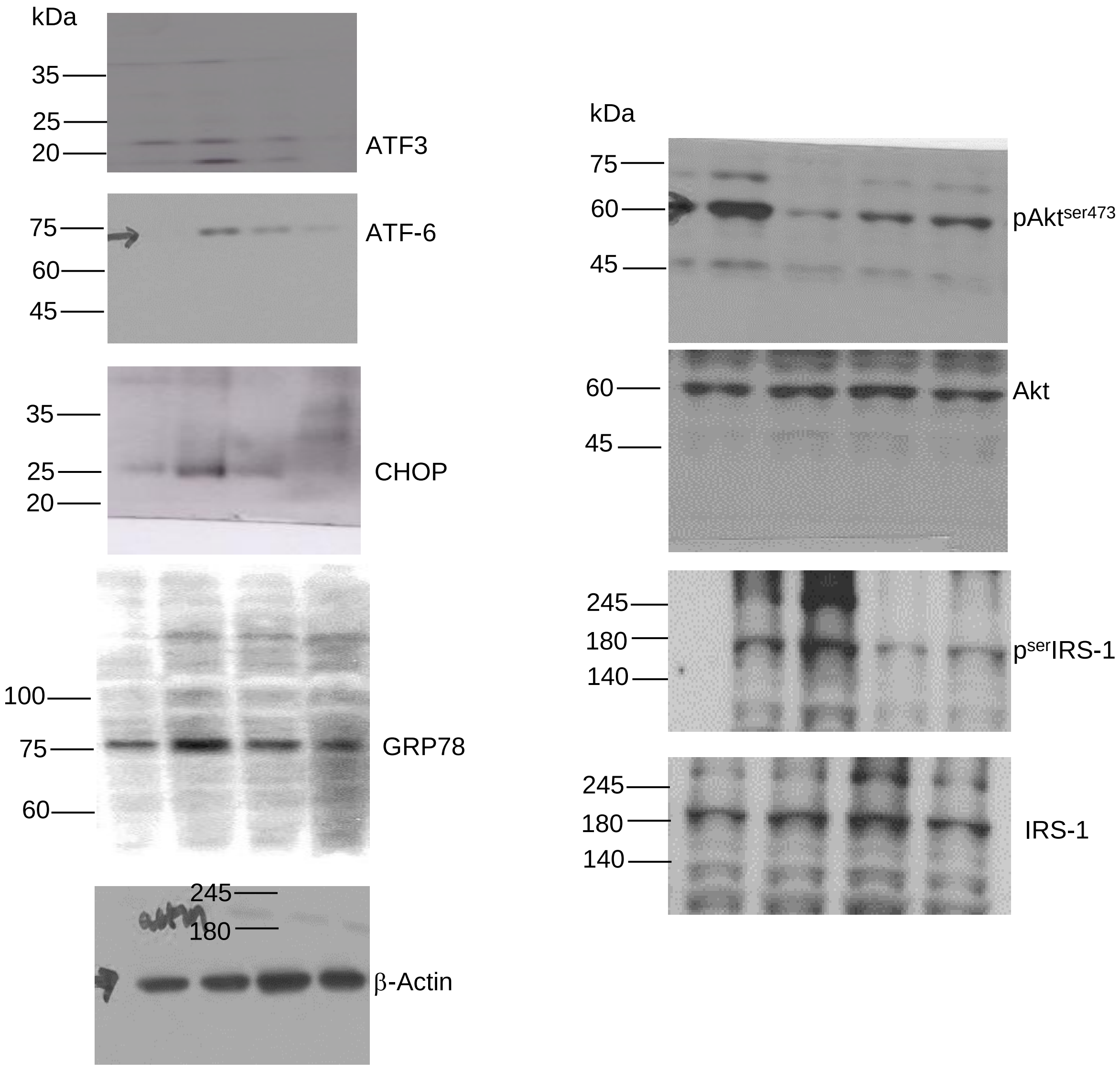

Supplementary Figure 6. Uncropped scans of western blot displayed in Fig. 8a and e

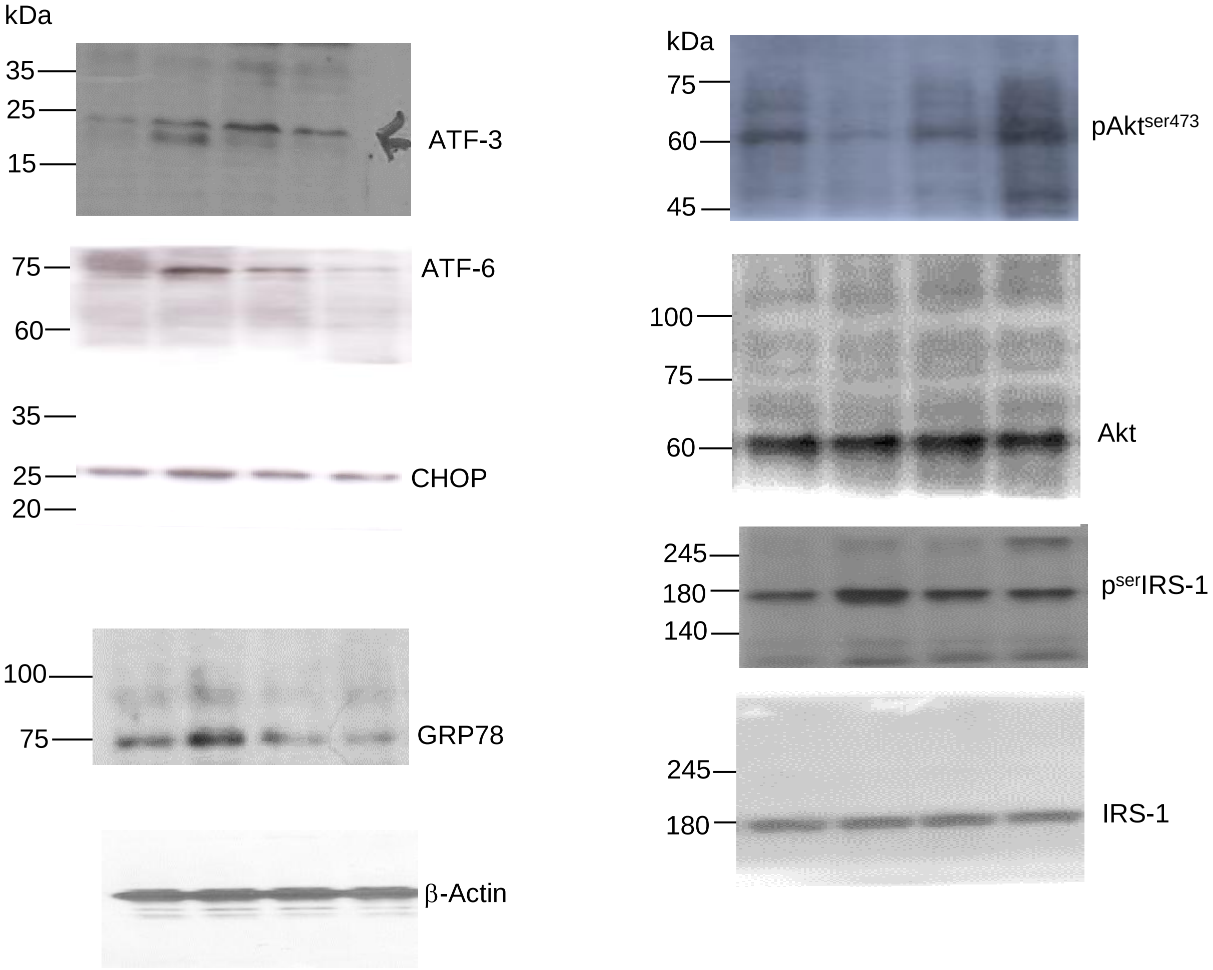

Supplementary Figure 7. Uncropped scans of western blot displayed in Fig. 8f and j

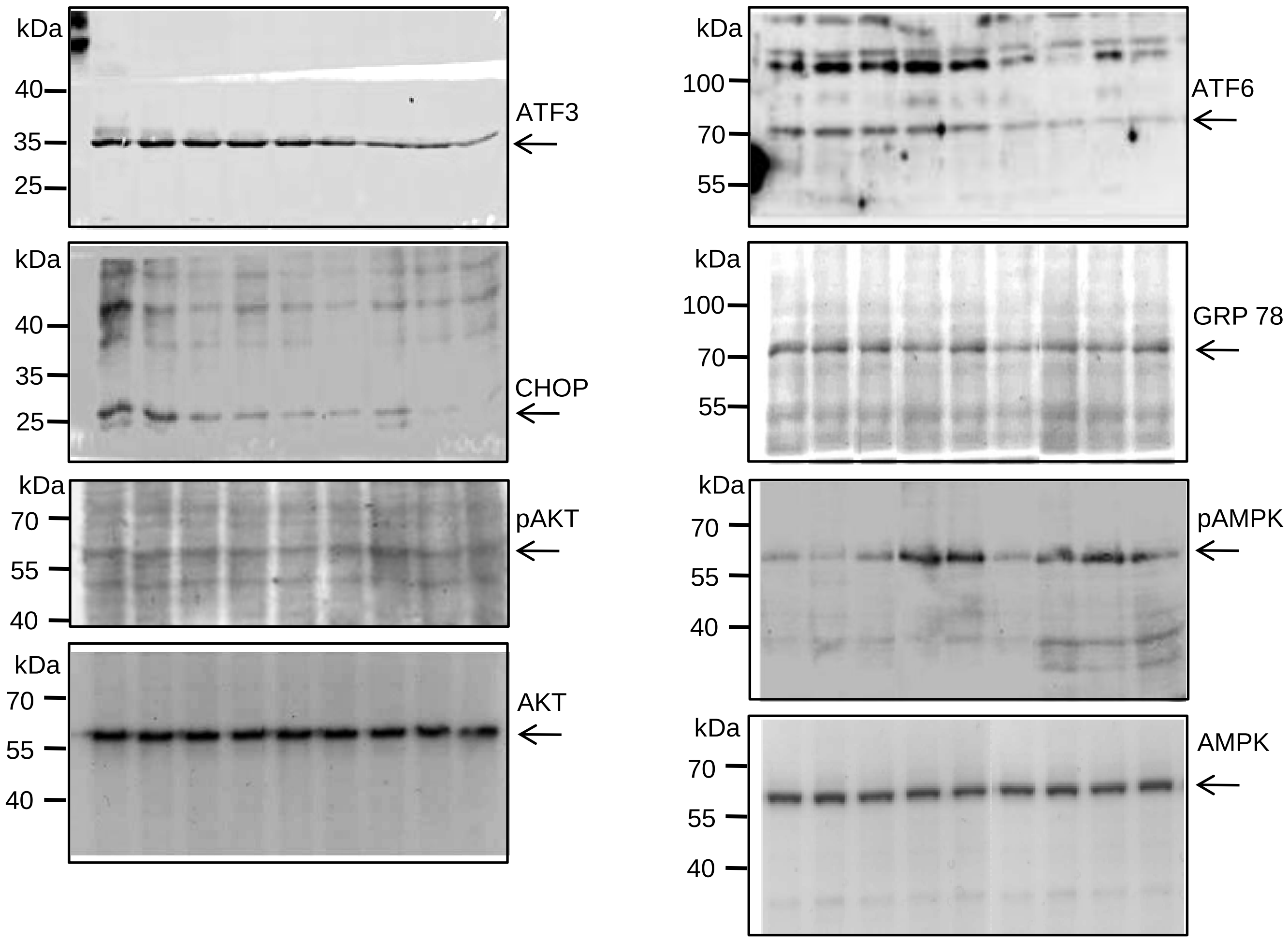

Supplementary Figure 8. Uncropped scans of western blot displayed in Fig. 9c (liver)

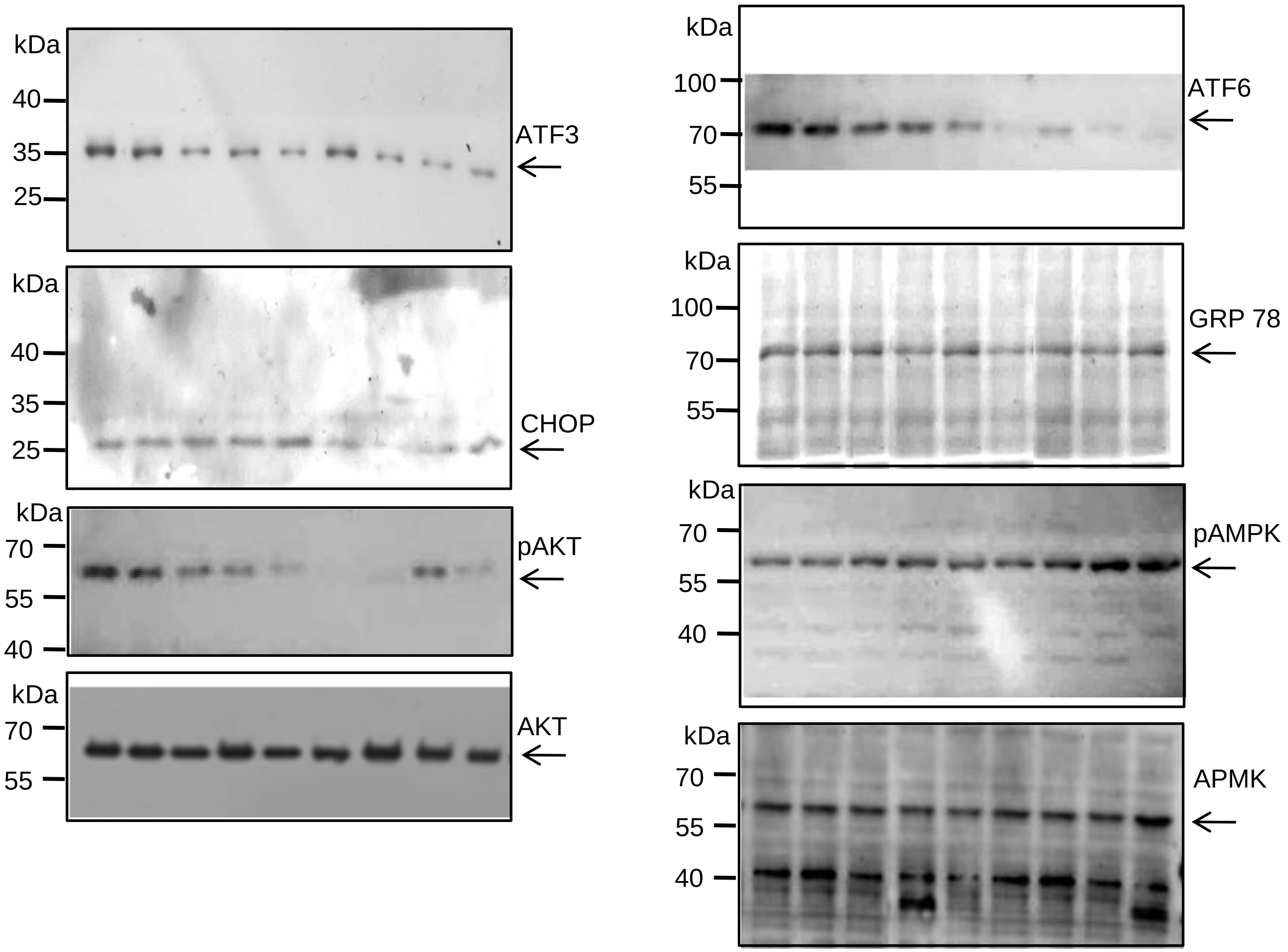

Supplementary Figure 9. Uncropped scans of western blot displayed in Fig. 9c (sWAT)
